# Effectiveness of Digital Health Interventions on Rehabilitation Outcomes in Patients After Total Knee Arthroplasty: Systematic Review and Network Meta-Analysis

**DOI:** 10.2196/101277

**Published:** 2026-11-13

**Authors:** Wenhua Zhang, Jingqi Mei, Xun Li, Jun Liu, Keliang Chen, Dianbo Zhang

**Affiliations:** 1 College of Physical Education and Health, Guangxi Normal University, Guilin, Guangxi, China; 2 Department of Nursing, College of Medicine, Hunan Normal University, Changsha, Hunan, China; 3 College of Sport and Health, Shandong Sport University, Jinan, Shandong, China; 4 Sports Science Research Institute, Shandong Sport University, Jinan, Shandong, China; 5 School of Sport Communication and Information Technology, Shandong Sport University, No. 10600, Shiji Avenue, Jinan, Shandong, 250102, China, +86 18653113037

**Keywords:** digital health interventions, total knee arthroplasty, postoperative rehabilitation, network meta-analysis, knee function

## Abstract

**Background:**

Total knee arthroplasty (TKA) is the most effective treatment for end-stage knee osteoarthritis, but some patients continue to experience persistent postoperative pain, stiffness, and impaired functional recovery. Digital health interventions are increasingly used in rehabilitation after TKA, but their comparative effectiveness remains unclear.

**Objective:**

This study aimed to evaluate the effects of different digital health interventions on rehabilitation outcomes in patients after TKA and to compare and rank these interventions in terms of pain relief, active knee flexion range of motion (ROM), and knee function to provide evidence for selecting optimal rehabilitation strategies.

**Methods:**

We searched PubMed, Embase, Cochrane Library, Web of Science, Google Scholar, Scopus, CINAHL, MEDLINE (via OvidSP), CNKI, Wanfang, and VIP to identify randomized controlled trials (RCTs) meeting the inclusion criteria. The RoB 2 tool (Risk of Bias 2; Cochrane) was used to assess risk of bias, and the confidence in network meta-analysis (CINeMA) framework was used to evaluate certainty of evidence. Traditional meta-analysis and network meta-analysis (NMA) were performed using Stata (version 17.0; StataCorp LLC). A random-effects model (DerSimonian-Laird method for estimating *τ²*) was consistently used across all analyses. For outcomes measured with the same or convertible scales, mean differences (MDs) with 95% CIs were used to pool effect sizes. Surface under the cumulative ranking curve (SUCRA) values were calculated to summarize the ranking probabilities.

**Results:**

A total of 36 RCTs involving 3067 patients after TKA were included. NMA results showed that compared with conventional rehabilitation (CR), for active flexion ROM, rehabilitation robot (RR; MD 8.96, 95% CI 5.86-12.05), mobile app–based telerehabilitation (MAT; MD 6.36, 95% CI 2.81-9.91), and virtual reality (VR; MD 5.92, 95% CI 2.94-8.90) were significantly superior to CR; for the Hospital for Special Surgery knee score, VR (MD 9.53, 95% CI 5.18-13.88), intelligent rehabilitation system-based telerehabilitation (MD 6.90, 95% CI 2.35-11.44), and RR (MD 5.32, 95% CI 1.61-9.04) were significantly superior to CR; for pain, RR and MAT had relatively high ranking probabilities, but the 95% CIs of most comparisons included 0; for the Western Ontario and McMaster Universities Osteoarthritis Index and Timed Up and Go test, only internet videoconference-based telerehabilitation and VR-based telerehabilitation, respectively, were significantly superior to CR. The certainty of evidence for most comparisons was low or very low.

**Conclusions:**

Digital health interventions may improve pain, active knee flexion ROM, and knee function in patients after TKA. In the SUCRA ranking, RR, VR, and MAT showed favorable ranking probabilities; however, because the network was predominantly star-shaped and the rankings were derived largely from indirect comparisons, these findings should be interpreted as suggestive rather than definitive. Further high-quality studies with direct head-to-head comparisons are needed.

## Introduction

In recent years, with the acceleration of population aging and changes in lifestyle, the global disease burden of knee osteoarthritis (KOA) has continued to increase. The latest research data show that the number of patients with KOA worldwide reached approximately 374.7 million in 2021, and the age-standardized prevalence has increased by more than 200% since 1990 [1]. In addition, KOA is also a common degenerative arthritis condition among athletes, for whom repeated knee joint injuries are an important risk factor [2].

Total knee arthroplasty (TKA) is the most effective surgical treatment for end-stage KOA. Its postoperative clinical efficacy is well established, patient satisfaction is high, and the number of surgeries has increased over time. For example, in a Chinese study with more than 20 years of follow-up, the prosthesis survival rates at 10, 15, and 20 years were 93.6%, 92.4%, and 89.8%, respectively. Although some prostheses showed slight functional decline after 20 years, overall patient satisfaction remained high [3]. However, even though most patients benefit after surgery, some studies have reported that approximately 13%‐44% of patients with TKA experience long-term persistent postoperative pain, a proportion that highlights the importance of postoperative rehabilitation and management challenges [4]. In addition, some patients may also experience postoperative joint stiffness and poor functional recovery, further affecting quality of life [5].

In this context, digital health technologies, including virtual reality (VR), remote home rehabilitation, wearable devices, and rehabilitation robots (RRs), have shown potential for integrating traditional rehabilitation with modern technology. Some studies have shown that, within 1 month after surgery, patients with TKA who use an intelligent monitoring system for home rehabilitation experience greater improvements in knee function, isometric knee extensor strength, and quality of life than those receiving traditional rehabilitation [6]. Studies have shown that wearable devices plus remote monitoring can continuously and passively collect data on patient activity (eg, step count), knee range of motion (ROM), adherence to home training, and pain, and have been proven to be feasible and highly acceptable to patients, enabling health care professionals to obtain more complete and real-time recovery trajectory data for personalized adjustment of rehabilitation plans [7]. A remote rehabilitation program based on smart wearable devices (such as BPMpathway [270 Vision Limited]) has also shown positive effects in Chinese patients with TKA. Compared with traditional home rehabilitation, patients in this group showed better improvement in postoperative knee ROM, functional recovery, and quality of life [8].

Previous systematic reviews and meta-analyses have separately evaluated the effects of different digital health technologies in rehabilitation after TKA. For example, the systematic review and meta-analysis by Gazendam et al [9] summarized the application of VR rehabilitation after TKA and found potential advantages in some functional outcomes; the meta-analysis by Yoo et al [10] evaluated the effects of robot-assisted rehabilitation in patients after TKA or total hip arthroplasty; a systematic review published by Curzio et al [11] in 2025 evaluated 14 randomized controlled trials (RCTs) involving VR, exergames, and telerehabilitation, and the results showed that these digital interventions were noninferior or superior to conventional rehabilitation (CR) in pain relief and functional improvement. Recently, a meta-analysis specifically comparing mobile app–based digital rehabilitation with traditional rehabilitation included 8 studies, and the results showed that app-based rehabilitation was superior to traditional rehabilitation in pain relief, Timed Up and Go (TUG), and the 10-meter walk test [12]; another systematic review published in 2025 evaluated the effects of smart device-assisted telerehabilitation versus CR after TKA [13]; and an umbrella review systematically synthesized evidence from multiple meta-analyses on the use of telerehabilitation after TKA [14].

However, existing systematic reviews and meta-analyses have the following limitations: first, most reviews focused on pairwise comparisons between a single digital rehabilitation mode (such as only VR, only robots, or only mobile apps) and CR and could not answer the clinical question of which digital rehabilitation mode is effective; second, some reviews treated different forms of telerehabilitation as a homogeneous category for pooling, ignoring differences in technological mechanisms; third, most existing studies lacked indirect comparisons among different digital modes and could not provide treatment ranking information.

Network meta-analysis (NMA) can, in the absence of direct head-to-head studies, use a common comparator as a bridge to perform indirect comparisons among different interventions and evaluate the relative effectiveness and probability rankings among multiple interventions. Compared with previous systematic reviews, the added value of this NMA is as follows: first, it simultaneously includes 8 digital rehabilitation modes, rather than being limited to a single mode; second, it performs indirect comparisons using a common comparator (CR) and ranks different digital modes; third, it uses the confidence in network meta-analysis (CINeMA) framework to assess certainty of evidence rather than reporting only statistical significance; fourth, it verifies the robustness of results through sensitivity analyses using alternative node classifications; and fifth, it conducts subgroup analyses by assessment time window and treatment-dose matching to explore sources of heterogeneity. These analytical dimensions were not covered by previous single-mode meta-analyses. Based on this, this study used NMA to explore the effects of different digital health interventions on rehabilitation outcomes in patients after TKA.

## Methods

### Protocol and Registration

This systematic review strictly followed the PRISMA (Preferred Reporting Items for Systematic Reviews and Meta-Analyses) 2020 guideline [15] and referred to the PRISMA-NMA extension statement to standardize the reporting of NMA-specific elements, including the description of network geometry, assessment of transitivity, inconsistency testing, and interpretation of ranking probabilities [15] (Checklist 1). The study protocol was registered on the International Prospective Register of Systematic Reviews (PROSPERO: CRD420251244837).

### Search Strategy

Two researchers began searching in September 2025, with the initial search cutoff on October 23, 2025. To ensure inclusion of the latest evidence, we updated the literature search on September 17, 2026, with the same databases, search terms, and inclusion and exclusion criteria as those used in the initial search. New studies meeting the inclusion criteria identified in the updated search were included in the analysis. We searched PubMed, Embase, Cochrane Library, Web of Science, Google Scholar, Scopus, CINAHL, MEDLINE (via OvidSP), CNKI, Wanfang, and VIP. In each database, we searched for RCTs on the effectiveness of digital health interventions for improving function in patients after TKA. The following search terms were used as search criteria: (AI OR VR OR wearable devices OR telemedicine OR telerehabilitation OR RR OR robot-assisted training OR intelligent rehabilitation system OR exergame OR exergaming OR serious game OR interactive gaming) AND (total knee replacement surgery OR TKR OR TKA) AND (RCT OR controlled clinical trial OR clinical trial OR randomized OR placebo OR randomly OR trial). The full search strategies for each database are provided in Multimedia Appendix 1.

### Inclusion and Exclusion Criteria

#### Inclusion Criteria:

Studies were eligible for inclusion if they met all of the following criteria:

1. Study type: all included studies had to be RCTs.
2. Participants: adult patients who had undergone TKA.
3. Comparator: the control group included physical therapy, routine care, balance training, continuous passive motion, manual guided rehabilitation, or health education. Although these interventions were heterogeneous, they shared the following core characteristics: all were components of standard rehabilitation after TKA, all aimed to improve joint ROM, muscle strength, and function, and none involved digital technology. Therefore, this study merged various forms of CR into the same node (CR), which served as the common comparator for all digital interventions.
4. Intervention: the experimental group received rehabilitation interventions based on digital health technology, including VR, VR technology-based telerehabilitation (VRT), mobile app–based telerehabilitation (MAT), internet videoconference-based telerehabilitation (IVT), intelligent rehabilitation system-based telerehabilitation (IRT), and RR, which could be used as the sole intervention or combined with other forms of rehabilitation. Studies were assigned to treatment nodes according to the following operational criteria:
  - VR: use of immersive or nonimmersive virtual reality technology, delivered face-to-face or in a clinical setting, not remotely, including AR. Interventions in this node shared the core characteristics of multisensory stimulation and interactive feedback.
  - VRT: VR technology delivered remotely via the internet or telecommunications. Interventions within this node shared the core characteristic of “virtual reality as the core content, delivered remotely.”
  - MAT: provided mainly through mobile apps on smartphones or tablets, with or without wearable sensors. Interventions within this node shared the core characteristic of “mobile application as the main delivery platform,” even if wearable sensors were also used.
  - IVT: provided mainly through internet-based videoconferencing. Interventions within this node shared the core characteristic of “real-time videoconferencing as the core communication method.”
  - IRT: provided through intelligent rehabilitation systems that integrate wearable sensors, cameras, or web platforms for remote monitoring and feedback. Although interventions within this node contained different technology combinations, they shared the core characteristic of “providing remote monitoring, feedback, and personalized adjustment through an integrated intelligent system.”
  - TFT: provided mainly through telephone follow-up. Interventions within this node shared the core characteristic of “telephone follow-up as the core communication method.”
  - RR: rehabilitation training using exoskeletons or end-effector robots. Interventions within this node shared the core characteristic of “robot-assisted training as the core intervention.”

The treatment-node classification in this study was prospectively specified during the study protocol design phase and finalized before data extraction; it was not adjusted based on data analysis results. Multicomponent interventions were classified according to the main technology defining the rehabilitation delivery method. Specifically: if the intervention was delivered mainly through a mobile app, it was classified as MAT, even if wearable sensors were also used; if the intervention was delivered mainly through an integrated intelligent system (such as camera-based motion capture or a web platform containing multiple sensors), it was classified as IRT; if the intervention was delivered mainly through videoconferencing, it was classified as IVT, even if a web portal was also used; if the intervention was delivered mainly through telephone, it was classified as TFT, even if printed materials were also provided. This method ensured that each study was assigned to the node reflecting its core therapeutic technology.

Outcomes: active flexion ROM, defined as the maximum angle a patient can achieve by actively flexing the knee; if the original study reported only passive ROM or total arc of motion, it was not included in the pooled analysis of ROM; visual analog scale (VAS), Hospital for Special Surgery (HSS) knee score, Western Ontario and McMaster Universities Osteoarthritis Index (WOMAC), numeric pain rating scale (NPRS), and Timed Up and Go (TUG) test.

At the same time, included studies needed to report or allow the extraction of rehabilitation-dose information for the experimental and control groups, including intervention frequency (sessions/week or sessions/day), duration per session (minutes), total number of sessions, intervention period (weeks), whether the digital intervention was added on top of CR, and total treatment contact time. If a study did not report sufficient dose information, it was marked as “dose unclear” and listed separately in the subgroup analyses.

#### Exclusion Criteria

Studies were eligible for exclusion if they met all of the following criteria:

(1) Duplicate publications, (2) participants who did not undergo primary, unilateral TKA as adults, (3) interventions not meeting inclusion criteria or comparators not meeting inclusion criteria, (4) outcomes not meeting the relevant inclusion criteria, (5) animal experiments, and (6) unclear study type or nonrigorous experimental design, such as non-RCTs, before-after self-control studies, case reports, and expert opinions.

### Literature Screening and Data Extraction

Retrieved records were imported into EndNote 20 (Clarivate) software, and duplicates were removed. Subsequently, 2 researchers screened the literature and extracted information according to the predefined inclusion and exclusion criteria. Disagreements were resolved through discussion with a third researcher. Characteristics of the included studies included the study reference, publication country, sample size, age, intervention, comparator, outcomes, supervision mode, supervisor, feedback type, feedback frequency, device and manufacturer, and rehabilitation dose information for the experimental and control groups (frequency, duration per session, total number of sessions, intervention period, whether the digital intervention was added, and total treatment contact time; Table 1).

At the same time, according to the relationship between the digital intervention and CR, included studies were divided into four intervention modes:

1. Digital intervention replacing CR: the digital intervention replaced CR, and the control group received only CR or face-to-face rehabilitation.
2. Digital intervention supplementing CR: the experimental group received CR+digital intervention, while the control group received only CR.
3. CR delivered digitally: the rehabilitation content was the same in both groups, but the experimental group received it through digital technology while the control group received it face-to-face.
4. Self-directed versus clinician-supervised: the experimental group received a self-directed digital intervention while the control group received clinician-supervised rehabilitation, or vice versa.

When a study met multiple modes simultaneously, it was assigned to a single primary category according to the following priority: (1) replacement > (2) supplementation > (3) digitally delivered > (4) self-directed versus clinician supervised.

For analysis, this study divided postintervention assessment time points into 3 assessment time windows according to common phases of rehabilitation after TKA: short-term (postoperative <6 weeks), medium-term (postoperative ≥6 weeks and <3 months), and long-term (postoperative ≥3 months). This time window division was based on the clinical trajectory of functional recovery after TKA: within 6 weeks after surgery, soft tissues are in the healing phase, joint swelling and stiffness are relatively high, and gait function recovery lags; at 3 months after surgery, the minimal clinically important difference (MCID) of the Oxford Knee Score (OKS) is 5.0 points (pain relief anchor) and 4.3 points (function anchor), and patients need to achieve approximately 4‐5 points of improvement to perceive clinical benefit; after 6 months, although the CID threshold of OKS changes over time, overall function tends to reach a stable plateau. Therefore, short-term corresponds to the early acute recovery phase, medium-term to the functional reconstruction phase, and long-term to the functional plateau phase [16,17].

For each outcome, data from the first assessment after the end of the intervention were extracted as the primary analysis time point. If a study reported multiple follow-up time points, data from the first assessment after the end of the intervention were extracted; if the study did not clearly report the end time of the intervention, the first assessment after the last intervention treatment was used. If a study reported only data during follow-up but not at the end of the intervention, the time point closest to the end of the intervention was extracted. If different outcomes in the same study had inconsistent assessment time points, data from the first postintervention assessment were extracted separately for each outcome, and the actual assessment time point corresponding to each outcome was recorded. All data were extracted as the means and SDs of baseline-to-postintervention change values; change scores and final values were not combined.

**Table 1.** Characteristics of the studies included (I).

Study	Country	Sample size (n)	Age (years), mean (SD)	Intervention	Comparator	Intervention mode	Experimental group dose	Control group dose	Dose matched	Device and manufacturer
Russell et al (2011) [18]	Australia	T^a^: 31 C^b^: 34	T: 66.2 (8.4) C: 69.6 (7.2)	IVT^c^	CR^d^	①^e^	45 minutes/session, 1 session/week, for 6 weeks	45 minutes/session, 1 session/week, for 6 weeks	Matched	Low-bandwidth videoconferencing via dial-up internet; specific device name mentioned Device name: Videoconferencing
Tousignant et al (2011) [19]	Canada	T: 21 C: 20	T: 66 (10) C: 66 (13)	IVT	CR	①	1 hour/session, 2 sessions/week, for 2 months	Specific dose not reported	Unclear	CODECs (videoconferencing codec) Manufacturer: Tandberg (now part of Cisco Systems)
Piqueras et al (2013) [20]	Spain	T: 72 C: 70	T: 73.3 (6.5) C: 73.3 (6.5)	VRT^f^	CR	①	60 minutes/session, 1 session/day, for 2 weeks	60 minutes/session, 1 session/day, for 2 weeks	Matched	Device name: Wireless 9DoF Kinematic Sensor Manufacturer: Shimmer
Moffet et al (2015) [21]	Canada	T: 104 C: 102	T: 65 (8) C: 67 (8)	IVT	CR	③^g^	45‐60 minutes/session, total 16 sessions, for 2 months	45‐60 minutes/session, total 16 sessions, for 2 months	Matched	Device name: IVT system Manufacturer: Telefónica
Chen et al (2016) [22]	China	T: 94 C: 93	T: 66.18 (3.59) C: 67.05 (4.05)	CR+TFT^h^	CR	②^i^	Total 12 weeks	1 hour/day at home, total 12 weeks	Experimental group higher	Telephone follow-up, no specific device involved
Jin et al (2018) [23]	China	T: 33 C: 33	T: 77.3 (3.8) C: 75.0 (5.1)	CR+VR^j^	CR	②	30 minutes/session, 3 sessions/day, for 6 days	Passive knee flexion maintained for 20 seconds, relaxation 40 seconds, 3 sets/day, 30 repetitions/set	Experimental group higher	Device name: VR Rehabilitation System Manufacturer: Mide Technology Inc (Cangzhou City, Hebei Province, China)
Kotani et al (2020) [24]	Japan	T: 12 C: 10	T: 77.3 (3.8) C: 75.0 (5.1)	CR+RR^k^	CR	②	Started on postoperative day 5, 50 repetitions every 2 days	Perform 50 active knee flexion repetitions every 2 days	Experimental group higher	Device name: HAL-SJ^l^ Manufacturer: Cyberdyne Inc (Ibaraki Prefecture, Japan)
Prvu Bettger et al (2020) [25]	United States	T: 143 C: 144	T: 65.4 (7.7) C: 65.1 (9.2)	VRT	CR	①	A standardized intervention protocol lasting 12 weeks	Specific dose not reported	Unclear	Device name: VERA^m^ Manufacturer: Reflexion Health (headquartered in San Diego, CA)
Gianola et al (2020) [26]	Italy	T: 35 C: 39	T: 66.6 (8.7) C: 70.7 (8.5)	CR+VR	CR	②	60 minutes/day for 1 week	60 minutes/day for 1 week	Experimental group higher	Device name: VRRS^n^ Manufacturer: not specified
Yoon and Son (2020) [27]	South Korea	T: 15 C: 15	T: 72.26 (3.65) C: 71.86 (4.89)	CR+VR	CR	②	30 minutes/day, 5 sessions/week, for 4 weeks	Specific dose not reported	Experimental group higher	Device name: VR Glass Manufacturer: Hyundai Chemistry (South Korea)
Prabhu et al (2020) [28]	United States	T: 9 C: 9	66.3 (8.2)	CR+VR	CR	②	5 minutes/time	Specific dose not reported	Experimental group higher	Device name: HTC Vive Pro Manufacturer: HTC
Bäcker et al (2021) [29]	Germany	T: 20 C: 25	T: 62.95 (8.25) C: 66.27 (10.57)	CR+MAT^o^	CR	②	5 minutes/session, 3‐5 sessions/day, for 6 weeks	Specific dose not reported	Experimental group higher	Device name: GenuSport (GenuSport Knietrainer) Developer/Institution: Hannover Medical School, Germany. Equipment composition: includes 3 piezoresistive force sensors, connected to a tablet mobile app
Pournajaf et al (2022) [30]	Italy	T: 29 C: 27	T: 68.31 (8.12) C: 71.07 (5.75)	CR+VR	CR	②	45 minutes/session, 5 sessions/week, for a total of 15 sessions	45 minutes/session, 5 sessions/week, for a total of 15 sessions	Experimental group higher	Device name: VRRS Manufacturer: Khymela Group (Noventa Padovana, Italy)
Mrotzek et al (2022) [31]	Germany	T: 17 C: 16	T: 58.13 (5.72) C: 59.31 (6.89)	RR	CR	③	30‐45 minutes/session, 2 sessions/day, for 9 days	30‐45 minutes/session, 2 sessions/day, for 9 days	Matched	Device name: HAL-SJ Manufacturer: Cyberdyne Inc (Headquartered in Tsukuba City, Japan)
Cai et al (2023) [32]	China	T: 40 C: 42	T: 66.98 (4.84) C: 65.55 (5.30)	CR+RR	CR	②	30 minutes/session, 2 sessions/day	10‐30 minutes/session, 2 sessions/day	Experimental group higher	Device name: Lokomat System Manufacturer: Hocoma AG
Janhunen et al (2023) [33]	Finland	T: 21 C: 25	T: 66.9 (3.1) C: 66.4 (4.5)	CR+VRT	CR	②	16 weeks, with progressive increases in frequency/duration/sets	16 weeks, with progressive increases in frequency/duration/sets	Experimental group higher	VR games, no specific device name mentioned
Shim et al (2023) [34]	South Korea	T: 27 C: 27	T: 68.25 (5.80) C: 72.96 (4.56)	CR+IRT^p^	CR	②	30 minutes/session, for 12 weeks	12 weeks, 3‐5 sets×10 repetitions/day	Experimental group higher	Device name: UINCARE Home+ Manufacturer: UINCARE Corp (Seoul, South Korea) System composition: rehabilitation program software, 3D depth camera, computer, and monitor
Yu et al (2023) [35]	South Korea	T: 12 C: 12	T: 69.54 (3.12) C: 68.39 (4.24)	VR (AR)^q^	CR	③	30 minutes/session, 3 sessions/week, for 4 weeks	30 minutes/session, 3 sessions/week, for 4 weeks	Matched	The specific device model and manufacturer were not reported
Zhao et al (2024) [36]	China	T: 50 C: 50	T: 65 (52-79) C: 65 (56-79)	IRT	CR	①	12 weeks	12 weeks	Matched	Device name: Vital Health Telerehabilitation System Manufacturer: Beijing Ailin Medical Technology Co, Ltd. System composition: patient-side mobile app, wearable sensors, and physician-side website
Nuevo et al (2024) [37]	Spain	T: 23 C: 22	T: 68.26 (5.42) C: 68.82 (4.41)	IRT	CR	③	4 weeks, 5 exercises/day, with up to 10 home visits	4 weeks, 5 exercises/day, with up to 10 home visits	Matched	Device name: ReHub Manufacturer: Bio-Sensing Solutions SL (DyCare) System composition: inertial motion sensors+web platform
Pua et al (2024) [38]	Singapore	T: 56 C: 58	T: 66.3 (7.5) C: 66.2 (6.9)	IRT	CR	①	45 minutes/session, for 10 weeks	At least 5 sessions of 45 minutes physical therapy within 10 weeks, with a maximum of 8 sessions	Mismatched	Microsoft Tablet Peripheral devices: digital goniometer, portable force plate system, and time-of-flight sensor Manufacturer: not specified
Bradbury et al (2024) [39]	United States	T: 95 C: 102	T: 70.2 (8.4) C: 70.2 (8)	IRT	CR	③	3 sessions/week, for 6 weeks	3 sessions/week, for 6 weeks	Mismatched	Device name: ERPM^r^ Manufacturer: Force Therapeutics (New York, NY)
Pérez-Maletzki et al [40]	Spain	T: 34 C: 31	T: 70 (10) C: 71 (7)	CR+MAT	CR	②	12 weeks, 5 days/week	12 weeks, 5 days/week	Experimental group higher	Smart mobile device, instant messaging app (WhatsApp, Telegram, and iMessage)
Jin et al (2017) [41]	China	T: 23 C: 27	64.4 (1.7)	CR+VR	CR	②	30 minutes/session, 3 sessions/day, for 6 days	Once daily, 40 minutes/session	Experimental group higher	VR equipment (specific model not mentioned) Manufacturer: Cangzhou Mide Technology Company
Sun and Sun (2017) [42]	China	T: 60 C: 60	T: 62.71 (8.97) C: 60.55 (9.26)	CR+TFT	CR	②	1 hour/session, for 12 weeks	Specific dose not reported	Experimental group higher	Telephone follow-up, no specific equipment involved
Li et al (2018) [43]	China	T: 39 C: 39	T: 71.82 (7.98) C: 72.97 (7.82)	CR+VR	CR	②	30 minutes/session, 3 sessions/day, for 2 weeks	Specific dose not reported	Experimental group higher	VR equipment (specific model not mentioned) Manufacturer: Zhuhai Zhuoru Technology Company
Feng et al (2018) [44]	China	T: 60 C: 60	T: 66.3 (9.3) C: 65.3 (8.4)	CR+VR	CR	②	30 minutes/session, 3 sessions/day, for 12 days	Specific dose not reported	Experimental group higher	VR equipment (specific model not mentioned) Provider: Cangzhou Mide Technology Company
Zhao et al (2018) [45]	China	T: 60 C: 60	T: 65.83 (8.91) C: 67.24 (9.32)	CR+IRT	CR	②	For 3 months	Specific dose not reported	Experimental group higher	Smart Wearable Assisted Rehabilitation System (specific name: NEO-SMART System) System composition: wearable smart ankle band, physician-side app, and patient-side app
Ding et al (2019) [46]	China	T: 49 C: 46	T: 60.7 (7.8) C: 62.2 (10.3)	CR+IRT	CR	②	For 2 weeks	Specific dose not reported	Experimental group higher	Wearable Joint Range of Motion Measurement Device Device structure and composition: each set contains 2 sensors
Zhang et al (2020) [47]	China	T: 35 C: 35	T: 68 (7) C: 71 (5)	CR+IRT	CR	②	Not reported	Specific dose not reported	Experimental group higher	Device name: NEO-GAIT “Qinbu” Smart Wearable Rehabilitation System Manufacturer: Beijing Shangda Medical Technology Co, Ltd Equipment composition: Sensors Patient-side app: “Gubingtong” app Physician-side app: “Gubingtong Doctor Version” app
Li et al (2021) [48]	China	T: 30 C: 30	T: 66.2 (6.7) C: 67.3 (8)	CR+MAT	CR	②	For 6 months	Specific dose not reported	Experimental group higher	App name: WeChat Manufacturer: Tencent, China
Zhou et al (2022) [49]	China	T: 41 C: 40	T: 58.9 (6.5) C: 57.6 (6.3)	CR+IRT	CR	②	Daily rehabilitation 4.6 (0.7) hours	Daily rehabilitation 4.6 (0.7) hours	Experimental group higher	Device name: Wearable Precision Rehabilitation System R&D team: independently developed by the research team at Southeast University. System composition: sensor nodes, sensor belt, wireless communication equipment, and mobile terminal
Li YS (2023) [50]	China	T: 39 C: 39	T: 67.9 (6.3) C: 68.9 (6.5)	CR+MAT	CR	②	12 weeks of remote guidance after discharge	Specific dose not reported	Experimental group higher	App name: WeChat Manufacturer: Tencent, China
Li (2023) [51]	China	T: 43 C: 43	T: 61.19 (7.06) C: 61.44 (7.68)	CR+RR	CR	②	Detailed protocol from 2 to 12 weeks postoperatively	Detailed protocol from 2 to 12 weeks postoperatively	Experimental group higher	Device name: Balance Disorder Rehabilitation Robot R&D institutions: Chinese People’s Liberation Army General Hospital, Beihang University, and Zhejiang University of Technology
Ma (2023) [52]	China	T: 30 C: 30	T: 6.93 (5.11) C: 65.83 (4.26)	CR+RR	CR	②	Detailed protocol from 1 to 4 weeks postoperatively	Detailed protocol from 1 to 4 weeks postoperatively	Experimental group higher	No device name or manufacturer information mentioned
Fan et al (2025) [53]	China	T: 50 C: 50	T: 64.8 (5.92) C: 64.8 (5.92)	CR+RR, 30 minutes/time, 3 times/week for 4 weeks	CR	②	30 minutes/session, 3 sessions/week, for 4 weeks	30‐45 minutes/session, 5 sessions/week, for 4 weeks	Experimental group higher	Robot-Assisted Balance Training System Developer: Chinese PLA General Hospital in collaboration with Beihang University

a T: experimental group.

b C: control group.

c IVT: internet videoconference-based telerehabilitation.

d CR: conventional rehabilitation.

e ①: digital intervention replacing conventional rehabilitation.

f VRT: VR-based telerehabilitation.

g ③: conventional rehabilitation delivered digitally.

h TFT: telephone follow-up-based telerehabilitation.

i ②: digital intervention supplementing conventional rehabilitation.

j VR: virtual reality.

k RR: rehabilitation robots.

l HAL-SJ: Single-Joint Hybrid Assistive Limb.

m VERA: Virtual Exercise Rehabilitation Assistant.

n VRRS: Virtual Reality Rehabilitation System.

o MAT: mobile app–based telerehabilitation.

p IRT: intelligent rehabilitation system-based telerehabilitation.

q AR: augmented reality.

r ERPM: Electronic Remote Perioperative Management Program.

When the original study did not directly report the SD of baseline-to-postintervention change values, this study used the following methods for data conversion and estimation: (1) if the study reported SE, SD was converted using the following formula [54,55]:


$$SD=SE\times \sqrt{n}$$

If the study reported 95% CI, different methods were used for conversion according to sample size. When the sample size was ≥100, the following was used:


$$SD=\sqrt{n}\times \frac{Upper-Lower}{3.92}$$

When sample size was <100, the following was used:


$$SD=\sqrt{n}\times \frac{Upper-Lower}{2\times t_{0.975,n-1}}$$

where Upper and Lower represent the upper and lower limits of the 95% CI, respectively, and *t* is the *t* value corresponding to the 2-sided 95% CI with degrees of freedom (n-1) [54,55].

If the study reported only pre- and postintervention means and their SDs, the following formula was used to estimate the SD of change, where the correlation coefficient r was estimated based on studies that directly reported the SD of change (*r*=0.5) [54,55]. In this study, *r*=0.5 was uniformly used for all outcomes, including pain, ROM, WOMAC, HSS, and TUG, and no different correlation coefficients were set for different outcomes.


$$SD_{change}=\sqrt{SD_{baseline}^{2}+SD_{final}^{2}-2r\times SD_{baseline}\times SD_{final}}$$

If the study reported only median and IQR, the following formula was used to estimate SD [55-57]:


$$SD\approx \frac{Q_{3}-Q_{1}}{1.35}$$

If the study reported only range, the following formula was used to estimate SD [55-57]:


$$SD\approx \frac{Maximum-Minimum}{4}$$

For studies containing multiple intervention subgroups that needed to be combined into a single comparison group, the following formula was used to calculate the pooled mean [54,55]:


$$Mean=\frac{n_{1}M_{1}+n_{2}M_{2}}{n_{1}+n_{2}}$$

The pooled SD was calculated using the following formula:


$$SD=\sqrt{\frac{(n_{1}-1)SD_{1}^{2}+(n_{2}-1)SD_{2}^{2}+\frac{n_{1}n_{2}}{n_{1}+n_{2}}(M_{1}-M_{2}{)}^{2}}{n_{1}+n_{2}-1}}$$

All data conversion and estimation processes were independently completed and cross-checked by 2 researchers.

### Risk of Bias Assessment

The Cochrane-recommended RoB 2 (Risk of Bias 2; Cochrane) tool was used to assess risk of bias in included RCTs. Two researchers independently performed the assessment, and disagreements were resolved through discussion or arbitration by a third researcher. RoB 2 judged the following 5 domains: (D1) randomization process, (D2) deviations from intended interventions, (D3) missing outcome data, (D4) measurement of the outcome, and (D5) selection of the reported result. Each domain and overall risk of bias were judged as “low risk,” “some concerns,” or “high risk.”

Given that blinding of participants and personnel is difficult in digital health interventions and that subjective outcomes (pain, WOMAC, and HSS) and objective outcomes (ROM and TUG) differ in sensitivity to blinding, this study assessed risk of bias separately for subjective and objective outcomes. At the same time, participant blinding and assessor blinding were recorded separately to distinguish their different impacts on risk of bias. For each study, it was clarified whether the analysis targeted an intention-to-treat (ITT) effect, and the analysis type (ITT, PP, or not reported) was recorded. Risk of bias assessment was performed separately for each outcome. For subjective and objective outcomes, assessment was based on the time point of the first assessment after the end of the intervention. The estimand of this study was the effect of assignment to the intervention, that is, analysis according to randomized groups. Because all experimental groups in included studies received the intervention, the effect of assignment and the effect of adherence were not substantially different in this study.

The signaling questions for high-risk judgments were as follows: D1 high risk: randomization sequence generation or allocation concealment methods were inadequate, or the randomization method was completely unreported and selection bias could not be ruled out; D2 high risk: there was obvious deviation from the intended intervention, and ITT analysis was not used, or only PP analysis was reported; D3 high risk: dropout rate >20%, or the proportion of missing data differed severely between groups, or dropout information/missing data handling methods were not reported; D4 high risk: assessor blinding was not reported, the outcome was subjective, and the measurement method might be affected by awareness of the intervention; D5 high risk: trial registration number was not reported, and there were signs of selective reporting.

### Statistical Analysis

Stata (version 17.0; StataCorp LLC) software was used for traditional meta-analysis and NMA. For outcomes measured with the same or convertible scales, mean differences (MDs) with 95% CI were used to pool effect sizes because MD has better clinical interpretability. Specifically, pain (VAS; 0‐100 mm) converted to a 0‐10 scale at 1 mm=0.1 points; NPRS (0‐10) used directly; if reported as 0‐100 points, converted to a 0‐10 scale at 1 point=0.1 points), ROM (degrees), TUG (seconds), and other outcomes could all be unified into the same or convertible scales; this study did not use standardized mean difference (SMD).

Given the expected clinical and methodological heterogeneity in interventions, rehabilitation protocols, populations, and assessment time points among included studies, this study prespecified a random-effects framework and did not switch to a fixed-effects model based on *I²* or *P* values. *I²* statistics and between-study variance τ² were used to assess heterogeneity. *τ²* was estimated using the DerSimonian-Laird method. Because each outcome was meta-analyzed separately in this study, *τ²* was estimated separately by outcome, and it was not assumed that all comparisons shared a single heterogeneity parameter. The *P* values for heterogeneity tests were reported only as descriptive information; *P*>.10 only indicates that the current data are insufficient to reject the homogeneity assumption and does not mean that there is no heterogeneity among studies. The *I²* values of 0%‐25% indicated negligible heterogeneity, 25%‐50% mild heterogeneity, 50%‐75% moderate heterogeneity, and >75% high heterogeneity.

Traditional meta-analysis used the metan command in Stata 17.0 (random-effects model, DL method for estimating *τ²*), and 95% prediction intervals (PrIs) were calculated (rfdist option) to reflect the range of effects that might be observed in future studies. NMA was fitted using the network command group in Stata 17.0 (frequentist framework), with CR as the reference treatment, and treatment effects were estimated using a multivariate normal approximation model. The mvmeta multivariate meta-analysis command was not used. Model fit was planned to be evaluated using node-splitting or global inconsistency tests; if the network did not form closed loops and the above tests could not be performed, the rationality of indirect comparisons was assessed through evaluation of the transitivity assumption. This study did not include multi-arm studies, so no special handling for multiarm studies was needed. Treatment effects were presented as the main results. Surface under the cumulative ranking curve (SUCRA) values were calculated and reported as supplementary information to represent ranking probabilities rather than definitive evidence of superiority. For each outcome, a rankogram was generated to show the probability of each intervention ranking first, second, third, etc.

In addition to statistical significance, this study also assessed the clinical significance of each outcome with reference to MCID values reported in previous literature. The MCID thresholds used were as follows: pain (VAS 0‐100 mm) 15 mm [58], NRS (0‐10) 2 points [58], ROM 9° [59], WOMAC total score 15 points [60], HSS 5.41 points [61], and TUG 1.4 seconds [62]. If the lower limit of the pooled effect size and its 95% CI exceeded the MCID, the effect was considered clinically meaningful. It should be noted that the MCID for TUG came from a hip osteoarthritis population [5] and may not be fully applicable to the TKA population; caution is needed in interpretation.

### Subgroup Analysis

Subgroup analyses were performed according to different intervention types to explore potential sources of heterogeneity. This study extracted data from the first assessment after the end of the intervention; therefore, strictly defined “follow-up duration” (the interval between the end of the intervention and assessment) was close to zero in most studies. Subgroup analyses were performed by assessment time window (short-term <6 weeks, medium-term ≥6 weeks and <3 months, long-term ≥3 months), publication language (Chinese vs non-Chinese), risk of bias (high vs low/some concerns), and technology type within the IRT node (wearable sensors vs cameras vs web platforms).

Subgroup analyses were also performed according to whether the total treatment dose in the experimental and control groups matched:

1. Dose matched: baseline rehabilitation doses in both groups were reported, and frequency, duration, number of sessions, and period were basically consistent; the digital intervention did not additionally increase total treatment exposure, or total doses in the 2 groups were comparable.
2. Experimental group higher: baseline rehabilitation doses in both groups were reported and basically consistent, but the experimental group had clearly higher total treatment exposure due to the additional digital intervention.
3. Experimental group lower: baseline rehabilitation doses in both groups were reported and basically consistent, but the digital intervention replaced part of CR, resulting in clearly lower total treatment exposure in the experimental group.
4. Unclear: at least one of the 2 groups did not report sufficient baseline rehabilitation dose information, making it impossible to judge whether baseline rehabilitation doses were consistent between groups or to determine the total dose relationship.
5. Mismatched: baseline rehabilitation doses in both groups were reported, but frequency, duration, number of sessions, and period were clearly inconsistent and could not be classified into (2) or (3).

For studies in which the control group did not report specific doses, the category “experimental group higher” was not assigned; instead, they were classified as “unclear” to avoid overestimating the impact of additional treatment dose.

### Publication Bias Assessment

When at least 10 studies were available for an outcome, comparison-adjusted funnel plots and Egger test were used to assess publication bias. For each outcome, a comparison-adjusted funnel plot was generated to visually inspect the symmetry of study points around the comparison-specific pooled effect. Egger test was used to statistically detect small-study effects, with *P*<.05 considered statistically significant.

### Assessment of the Transitivity Assumption

This study assessed the transitivity assumption by comparing the distribution of potential effect modifiers among treatment nodes, including participant age, intervention duration (short-term <6 weeks, medium-term ≥6 weeks and <3 months, and long-term ≥3 months), sample size, baseline pain score, baseline active flexion ROM, baseline WOMAC score, baseline HSS score, baseline TUG time, and unilateral versus bilateral TKA, postoperative intervention start time, inpatient versus home rehabilitation setting, type and intensity of CR, risk of bias distribution, and geographic region. If the distribution of these factors among nodes was similar, the transitivity assumption might hold; if significant differences existed, they were explained when interpreting the results. If the network did not form closed loops and node-splitting or global inconsistency tests could not be performed, transitivity assessment would serve as supplementary evidence for the validity of indirect comparisons.

### Certainty of Evidence (CINeMA)

The CINeMA framework was used to assess certainty of evidence for each comparison. This framework considers 6 domains: within-study bias, reporting bias, indirectness, imprecision, heterogeneity, and incoherence. Each domain was rated as no concerns, some concerns, or major concerns, and overall certainty was rated as high, moderate, low, or very low. If no closed loops were formed, incoherence could not be assessed, and a summary table was generated to present the ratings.

## Results

### Study Selection

A total of 8729 records were retrieved from PubMed, Embase, the Cochrane Library, Web of Science, Google Scholar, Scopus, CINAHL, MEDLINE (via OvidSP), CNKI, Wanfang, and VIP. After screening titles, abstracts, and full-text studies according to inclusion and exclusion criteria, 36 studies were ultimately included in the meta-analysis (Figure 1).

**Figure 1.**
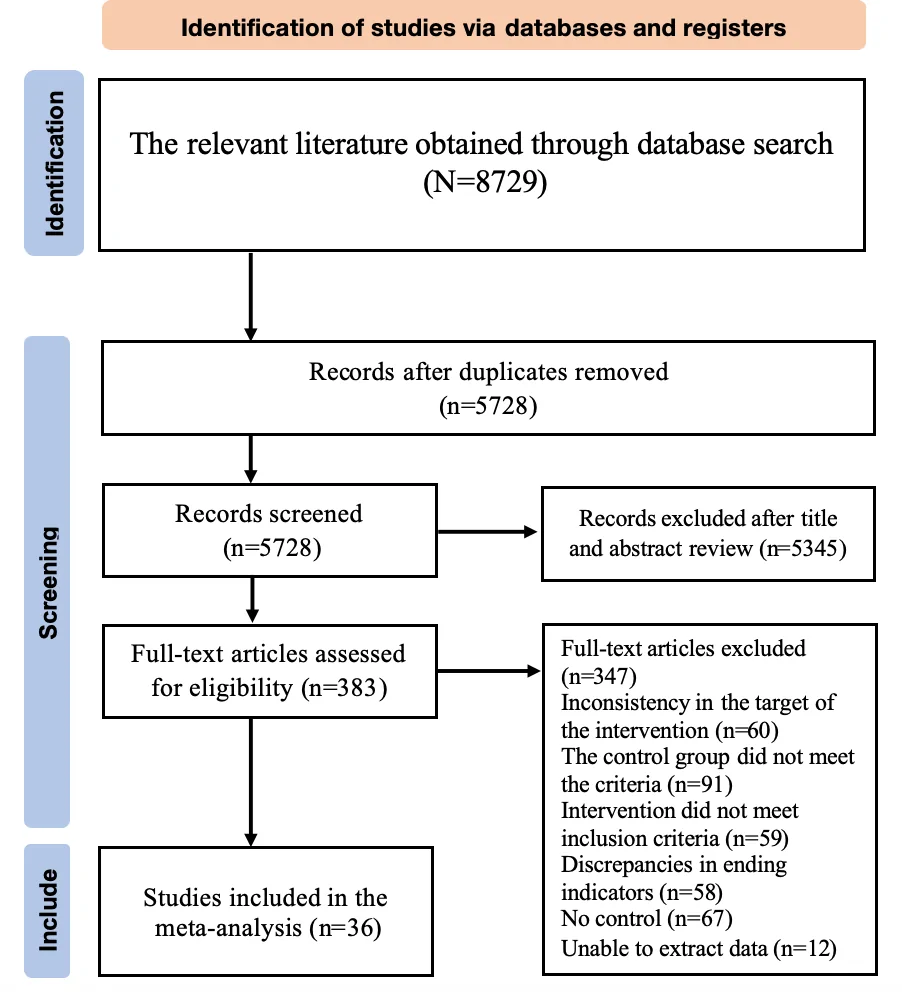
PRISMA (Preferred Reporting Items for Systematic Reviews and Meta-Analyses) flowchart.

### Characteristics of the Included Studies

This study included 36 articles [18-53] published between 2011 and 2026, encompassing 3067 participants (1552 in the experimental and 1515 in the control groups). Most studies were conducted in China (n=17) [22,23,32,36,41-53], followed by the United States (n=3) [25,28,39], South Korea (n=3) [27,34,35], Canada (n=2) [19,21], Italy (n=2) [26,30], Germany (n=2) [29,31], Spain (n=3) [20,37,40], and one each from Japan [24], Australia [18], Finland [33], and Singapore [38]. Interventions included IVT (n=3) [18,19,21], VRT (n=3) [20,25,33], TFT (n=2) [22,42], MAT (n=4) [29,40,48,50], IRT (n=9) [34,36-39,45-47,49], VR (n=8) [23,26-28,30,41,43,44], RR (n=6) [24,31,32,51-53], and augmented reality (AR; n=1) [35] (Table 1).

### Risk of Bias

The RoB 2 tool was used to assess the risk of bias separately for subjective and objective outcomes; detailed judgments for each domain are provided in Multimedia Appendix 2. Most studies were rated as “some concerns,” with only a few rated as “high risk.” For subjective outcomes, 9 studies were rated as having a low risk of bias, 20 as having some concerns, and 2 as having a high risk of bias [29,34]; both were rated as having a high risk of bias in D3, mainly because the dropout rate was >20% or the methods for handling missing data were not reported. For objective outcomes, 11 were low risk, 18 had some concerns, and 2 were high risk [34,47]; both due to D3.

### Safety Outcomes

Of 36 studies [18-53], 17 [21,25,26,30-34,36-40,43,45,46,50] reported at least one safety outcome. The most common was adverse events (17 studies), followed by discontinuation due to adverse events (5), readmission (3), device-related complications (2), and falls (1). Because definitions varied, reporting rates were low, and data were sparse, no quantitative pooling was performed. Prvu Bettger et al [25] reported that the fall rate in the virtual physical therapy group was slightly higher than that in the conventional group (19.4% vs 14.6%), but readmissions were fewer (12 vs 30). Nuevo et al [37] reported 1 case of moderate-severity functional overload related to ReHub. Gianola et al [26] reported a higher dropout rate in the VR group due to discomfort/nonacceptance of the device. Overall, no clear evidence of a high safety risk was associated with digital health technologies, but the safety evidence remains insufficient. See Table 2 for details.

**Table 2.** Characteristics of the studies included (II).

Study	Supervision mode	Supervisor	Feedback type	Feedback frequency	Postintervention assessment time point (postoperative)	Assessment time window	Outcomes	Safety outcomes	Key safety results
Russell et al (2011) [18]	Remote synchronous supervision	Physiotherapist	Visual + auditory (videoconference)	Real-time	6 weeks postoperatively	Medium-term	VAS^a^, ROM^b^, WOMAC^c^, and TUG^d^	NR^e^	—^f^
Tousignant et al (2011) [19]	Remote synchronous supervision	Physiotherapist	Visual + auditory (videoconference)	Real-time	At the end of the intervention (approximately 2 months postoperatively)	Medium-term	TUG	NR	—
Piqueras et al (2013) [20]	Remote synchronous supervision	Physiotherapist	Visual + auditory + biofeedback	Real-time	2 weeks postoperatively	Short-term	VAS, ROM, and TUG	NR	—
Moffet et al (2015) [21]	Remote synchronous supervision	Physiotherapist	Visual + auditory (videoconference)	Real-time	At the end of the intervention (approximately 2 months postoperatively)	Medium-term	ROM	AE^g^	No serious adverse events in either group
Chen et al (2016) [22]	Remote asynchronous supervision	Nurse	Auditory (telephone)	Regular	12 weeks postoperatively	Long-term	VAS and ROM	NR	—
Jin et al (2018) [23]	Face-to-face supervision	Researcher	Visual + auditory + gamified feedback	Real-time	2 weeks postoperatively	Short-term	VAS, WOMAC, and HSS^h^	NR	—
Kotani et al (2020) [24]	Face-to-face supervision	Physiotherapist	Visual + auditory + biofeedback	Real-time	10 days postoperatively	Short-term	VAS and ROM	NR	—
Prvu Bettger et al (2020) [25]	Remote asynchronous supervision	Physiotherapist	Visual + auditory + 3D biofeedback	Real-time	12 weeks postoperatively	Long-term	NPRS^i^	AE, falls, and readmission	Virtual PT^j^ group falls 19.4% versus conventional 14.6%, readmission 12 versus 30, and no device-related serious AE
Gianola et al (2020) [26]	Face-to-face supervision	Physiotherapist	Visual + auditory + biofeedback	Real-time	At discharge (approximately 10 days postoperatively)	Short-term	VAS, ROM, and WOMAC	AE and discontinuation due to AE^k^	The VR group had a higher dropout rate, mainly due to discomfort/nonacceptance of the VR device
Yoon and Son (2020) [27]	Face-to-face supervision	Physiotherapist	Visual + auditory + gamified feedback	Real-time	4 weeks postoperatively	Short-term	WOMAC and TUG	NR	—
Prabhu et al (2020) [28]	Face-to-face supervision	Researcher	Visual + auditory + biofeedback	Real-time	Immediately postoperatively	Short-term	VAS	NR	—
Bäcker et al (2021) [29]	Remote asynchronous supervision	Physician	Visual + gamified feedback	Real-time	6 weeks postoperatively	Medium-term	VAS	NR	—
Pournajaf et al (2022) [30]	Face-to-face supervision	Physiotherapist	Visual + auditory + biofeedback	Real-time	At the end of the intervention (approximately 3 weeks postoperatively)	Short-term	VAS and TUG	AE	No treatment-related adverse events
Mrotzek et al (2022) [31]	Face-to-face supervision	Physiotherapist	Visual + auditory + biofeedback	Real-time	At discharge (approximately 9 days postoperatively)	Short-term	ROM	AE and discontinuation	Complications; 1 case withdrew due to noncompliance and 1 case of nondevice-related infection
Cai et al (2023) [32]	Face-to-face supervision	Physiotherapist	Visual + auditory + biofeedback	Real-time	Day of discharge (approximately 9‐13 days postoperatively)	Short-term	ROM and HSS	AE	No adverse events in robot group
Janhunen et al (2023) [33]	No supervision (self-directed)	—	Visual + auditory + gamified feedback	Real-time	4 months postoperatively	Long-term	VAS, ROM, and TUG	AE	No intervention-related adverse events
Shim et al (2023) [34]	Remote asynchronous supervision	Physician	Visual + auditory + AR feedback	Real-time	12 weeks postoperatively	Long-term	NPRS, ROM, and WOMAC	AE and discontinuation due to AE	No serious adverse events; DR group had more dropouts due to discomfort/technical issues
Yu et al (2023) [35]	Face-to-face supervision	Physiotherapist	Visual + auditory + AR feedback	Real-time	4 weeks postoperatively	4 weeks postoperatively	VAS and ROM	NR	—
Zhao et al (2024) [36]	Remote asynchronous supervision	Physician	Visual + auditory + sensor feedback	Real-time	12 weeks postoperatively	Long-term	VAS, ROM, and WOMAC	AE and readmission	No readmission in either group; 1 case of superficial wound infection in each group
Nuevo et al (2024) [37]	Remote asynchronous supervision	Physiotherapist	Visual + auditory + animated feedback	Real-time	4 weeks postoperatively	Short-term	VAS, ROM, WOMAC, and TUG	AE, device-related complications, and discontinuation due to AE	ReHub group: 1 case of moderate-severe functional overload; no serious device-related AE
Pua et al (2024) [38]	Remote asynchronous supervision	Research team	Visual + auditory	Each training session	12 weeks postoperatively	Long-term	NPRS and ROM	AE	Serious adverse events similar between groups
Bradbury et al (2024) [39]	Remote asynchronous supervision	Physiotherapist	Visual + auditory + 3D feedback	Real-time	6 weeks postoperatively	Medium-term	NPRS, ROM, and TUG	AE and discontinuation due to AE	ROM/gait problems crossed over to OPT^l^
Pérez-Maletzki et al (2026) [40]	Remote asynchronous supervision	Researcher	Visual + auditory (chat messages)	Regular	3 months postoperatively	Long-term	VAS, ROM, and WOMAC	AE and discontinuation due to AE	No directly related adverse events; reasons for dropout included device malfunction and comprehension difficulties
Jin et al (2017) [41]	Face-to-face supervision	Researcher	Visual + auditory + gamified feedback	Real-time	6 days postoperatively	Short-term	VAS	NR	—
Sun and Sun (2017) [42]	Remote asynchronous supervision	Rehabilitation therapist	Visual (video) + auditory	Regular	12 weeks postoperatively	Long-term	ROM	NR	—
Li et al (2018) [43]	Face-to-face supervision	Researcher	Visual + auditory + gamified feedback	Real-time	2 weeks postoperatively	Short-term	VAS and HSS	AE	No DVT^m^; observation group had lower DVT incidence than control group
Feng et al (2018) [44]	Face-to-face supervision	Researcher	Visual + auditory + gamified feedback	Real-time	12 days postoperatively	Short-term	ROM and HSS	NR	—
Zhao et al (2018) [45]	Remote asynchronous supervision	Physician	Visual + auditory (App+3D video)	After each training session	3 months postoperatively	Long-term	ROM, WOMAC, and HSS	AE	No periprosthetic infection, DVT, or periprosthetic fracture
Ding et al (2019) [46]	Remote asynchronous supervision	Physician	Visual (App data)	After each training session	12 weeks postoperatively	Long-term	WOMAC and HSS	AE	No periprosthetic infection, DVT, or periprosthetic fracture
Zhang et al (2020) [47]	Remote asynchronous supervision	Physician	Visual + auditory (App)	Regular	12 weeks postoperatively	Long-term	ROM and TUG	NR	—
Li et al (2021) [48]	Remote asynchronous supervision	Physician	Visual + auditory (App)	Regular	6 months postoperatively	Long-term	VAS, WOMAC, and TUG	NR	—
Zhou et al (2022) [49]	Remote asynchronous supervision	Physician	Visual + auditory (App)	After each training session	6 months postoperatively	Long-term	ROM and HSS	NR	—
Li YS (2023) [50]	Face-to-face supervision	Physiotherapist	Visual + auditory + robot feedback	Real-time	4 weeks postoperatively	Short-term	VAS, ROM, WOMAC, and TUG	AE and readmission	No readmission, no DVT/PE, or other adverse events
Li (2023) [51]	Remote asynchronous supervision	Physician	Visual + auditory (WeChat video)	Regular	12 weeks postoperatively	Long-term	VAS, HSS, and TUG	NR	—
Ma (2023) [52]	Face-to-face supervision	Physiotherapist	Visual + auditory + weight-bearing feedback	Real-time	1 month postoperatively	Short-term	VAS, ROM, and HSS	NR	—
Fan et al (2025) [53]	Face-to-face supervision	Physiotherapist	Visual + auditory + balance feedback	Real-time	1 month postoperatively	Short-term	ROM, WOMAC, HSS, and TUG	NR	—

a VAS: visual analog scale.

b ROM: active flexion range of motion.

c WOMAC: Western Ontario and McMaster Universities Osteoarthritis Index.

d TUG: Timed Up and Go test.

e NR: not reported.

f Not applicable.

g AE: adverse events.

h HSS: Hospital for Special Surgery knee score.

i NPRS: numeric pain rating scale.

j PT: physical therapy.

k Discontinuation due to AE: discontinuation due to adverse events.

l OPT: outpatient physical therapy.

m DVT: deep vein thrombosis.

### Network Evidence

The network was mainly a star-shaped network centered on CR. Most trials compared “digital intervention+CR” with “CR alone,” with few direct comparisons between digital interventions. No closed loops were formed; therefore, node-splitting and global inconsistency tests were not applicable. VR and RR had the largest sample sizes. Pain was reported in 24 studies, ROM in 25, WOMAC in 13, HSS in 10, and TUG in 13. The network structure is shown in Figure 2.

**Figure 2.**
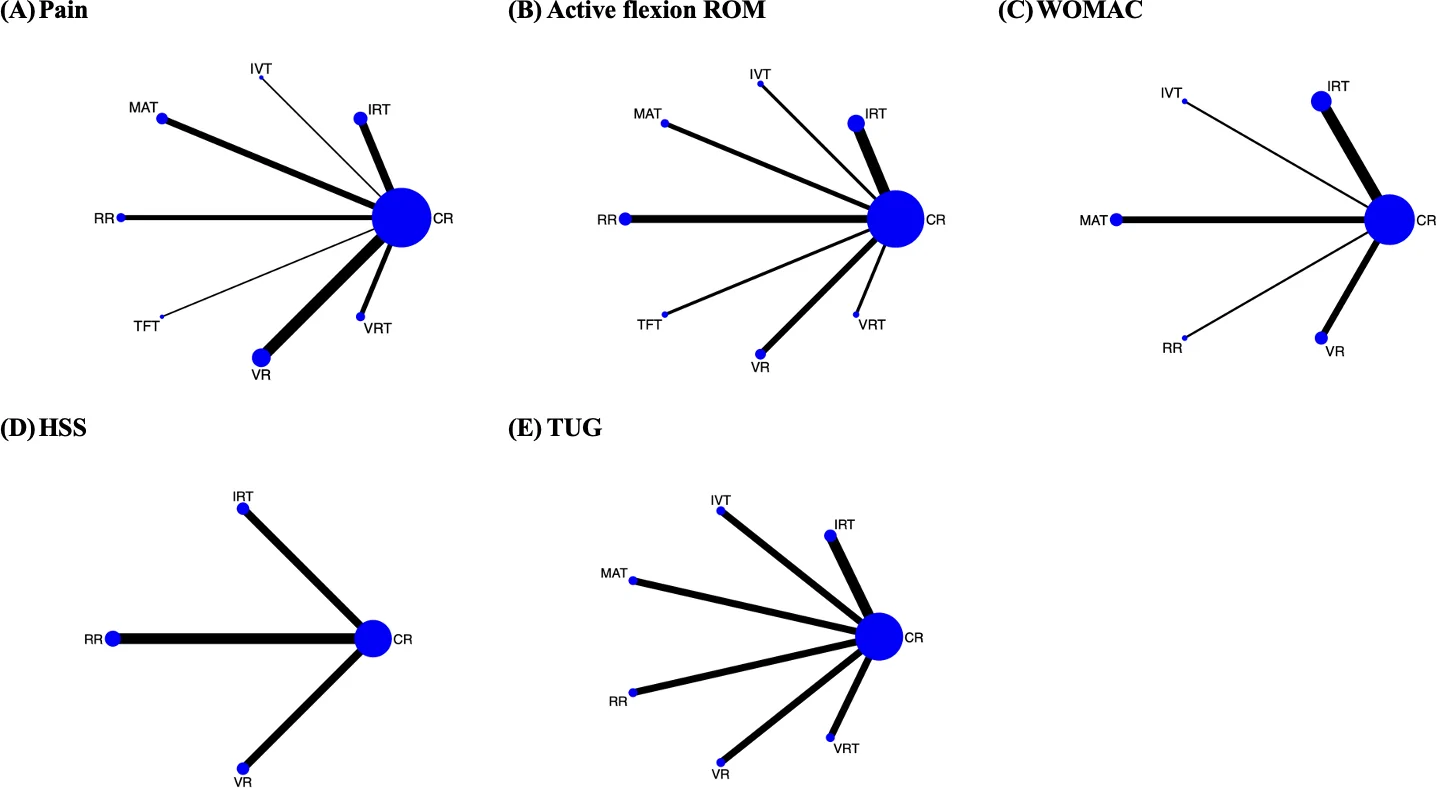
Network evidence plot. (A) Pain, (B) active flexion range of motion (ROM), (C) Western Ontario and McMaster Universities Osteoarthritis Index (WOMAC), (D) Hospital for Special Surgery (HSS) knee score, and (E) Timed Up and Go (TUG) test. CR: conventional rehabilitation; IRT: intelligent rehabilitation system-based telerehabilitation; IVT: internet videoconference-based telerehabilitation; MAT: mobile app–based telerehabilitation; RR: rehabilitation robots; TFT: telephone follow-up–based telerehabilitation; VR: virtual reality; VRT: VR-based telerehabilitation.

### NMA of Outcomes

### Overview

For pain, WOMAC, and TUG, negative MDs indicated benefit; for ROM and HSS, positive MDs indicated benefit. Results should be interpreted as the comprehensive effect of digital health interventions relative to CR, rather than as the additional benefit of a single “CR+digital intervention.”

#### Pain Scores

Compared with CR, RR (MD −0.81, 95% CI –1.73 to 0.11), MAT (MD −0.66, 95% CI –1.48 to 0.15), and VR (MD −0.58, 95% CI −1.23 to 0.07) showed greater pain relief, while IRT (MD 1.05, 95% CI 0.31-1.79) showed less, but most CIs included 0 (Figure 3A). Among digital interventions, RR, MAT, VR, and VRT were significantly superior to IRT. SUCRA rankings were, in order, RR (78.9%), MAT (72.7%), VR (70.0%), IVT (50.3%), TFT (46.8%), VRT (45.8%), CR (32.9%), and IRT (2.6%) (Figure 4A). RR had the highest probability of ranking first (35.3%), but the distribution was scattered (Figure 5A).

**Figure 3.**
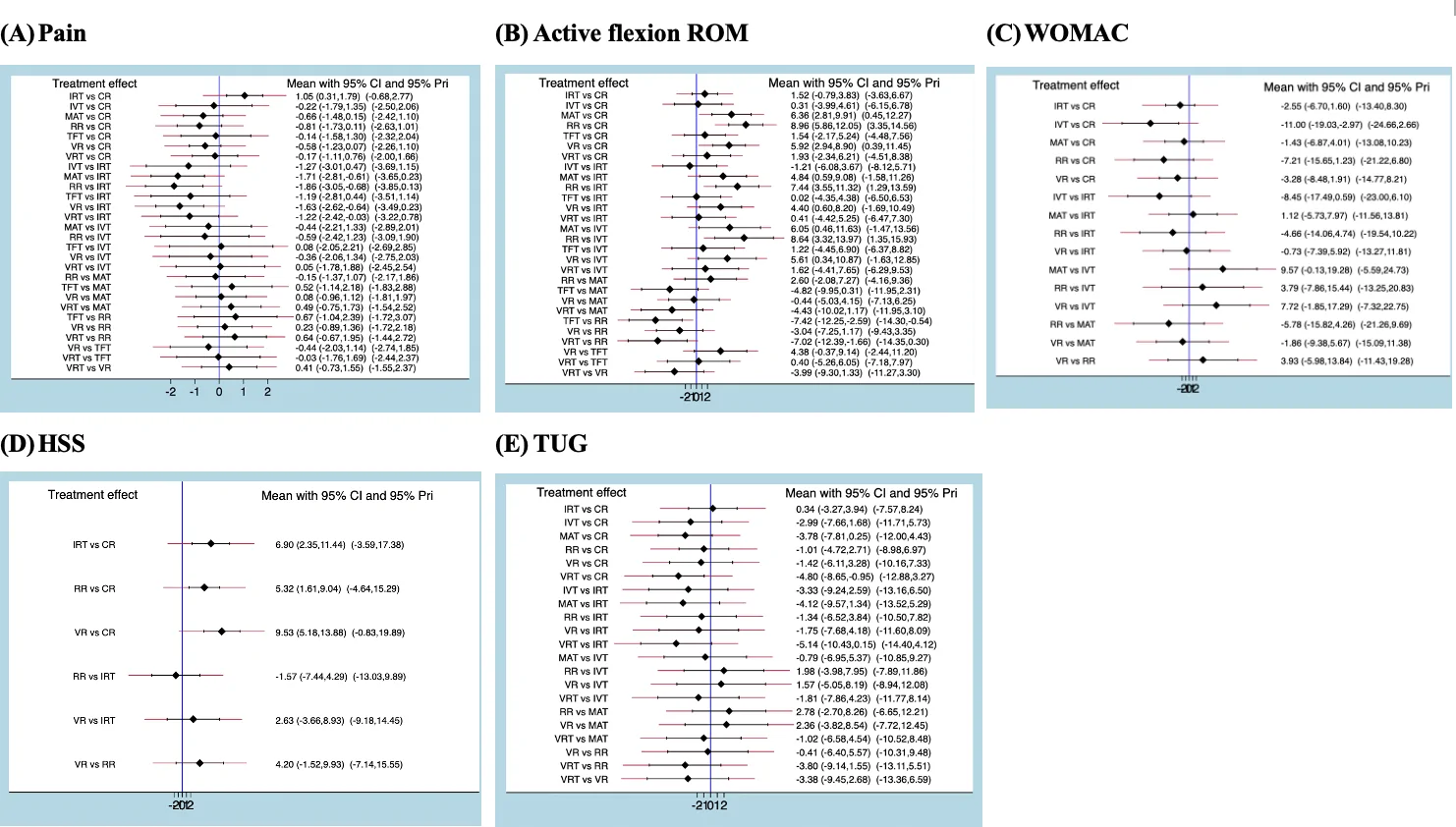
Forest plot of the network meta-analysis. (A) Pain, (B) active flexion range of motion (ROM), (C) Western Ontario and McMaster Universities Osteoarthritis Index (WOMAC), (D) Hospital for Special Surgery (HSS) knee score, and (E) Timed Up and Go (TUG) test. CR: conventional rehabilitation; IRT: intelligent rehabilitation system-based telerehabilitation; IVT: internet videoconference-based telerehabilitation; MAT: mobile app–based telerehabilitation; PrI: prediction interval; RR: rehabilitation robots; TFT: telephone follow-up-based telerehabilitation; VR: virtual reality; VRT: VR-based telerehabilitation.

**Figure 4.**
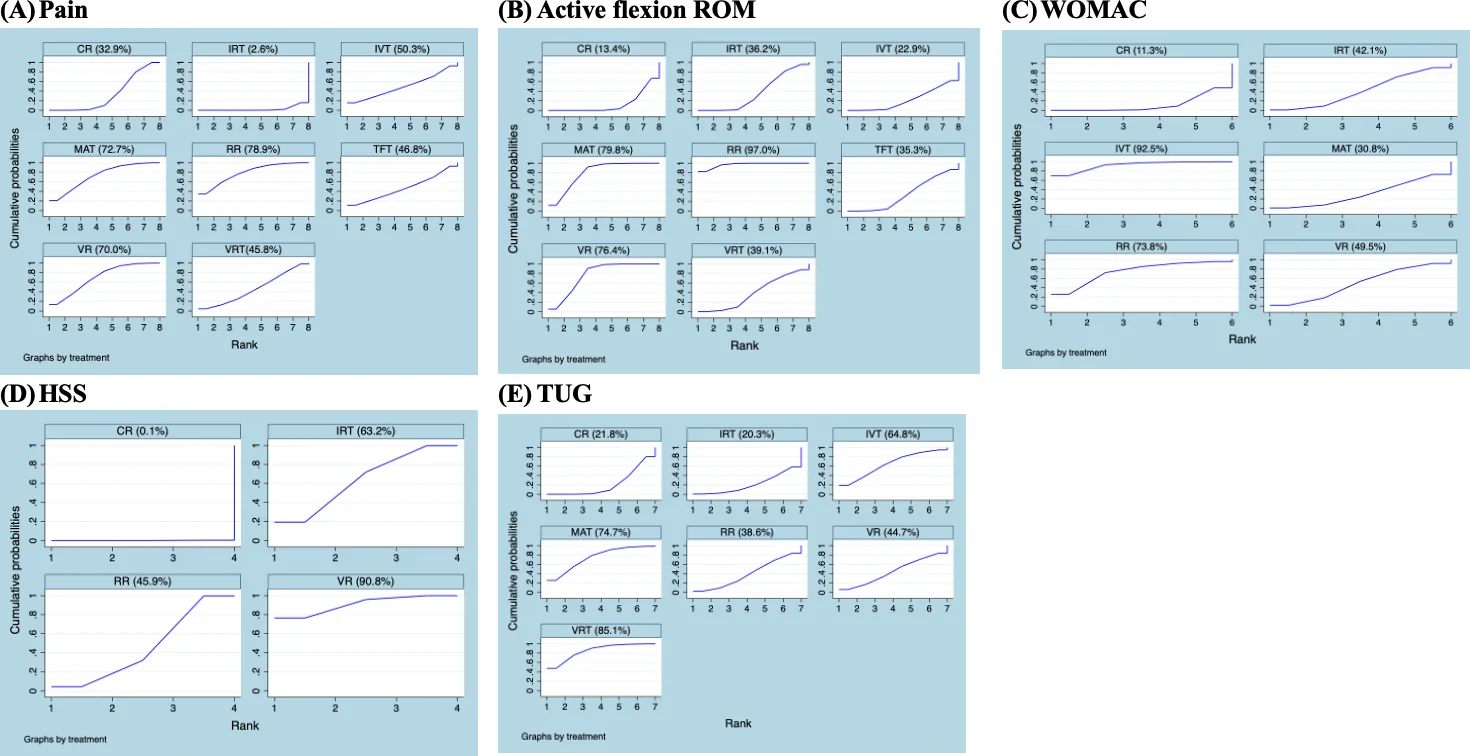
Cumulative probability ranking plot. (A) Pain, (B) active flexion range of motion (ROM), (C) Western Ontario and McMaster Universities Osteoarthritis Index (WOMAC), (D) Hospital for Special Surgery (HSS) knee score, and (E) Timed Up and Go (TUG) test. CR: conventional rehabilitation; IRT: intelligent rehabilitation system-based telerehabilitation; IVT: internet videoconference-based telerehabilitation; MAT: mobile app–based telerehabilitation; RR: rehabilitation robots; TFT: telephone follow-up-based telerehabilitation; VR: virtual reality; VRT: VR-based telerehabilitation.

**Figure 5.**
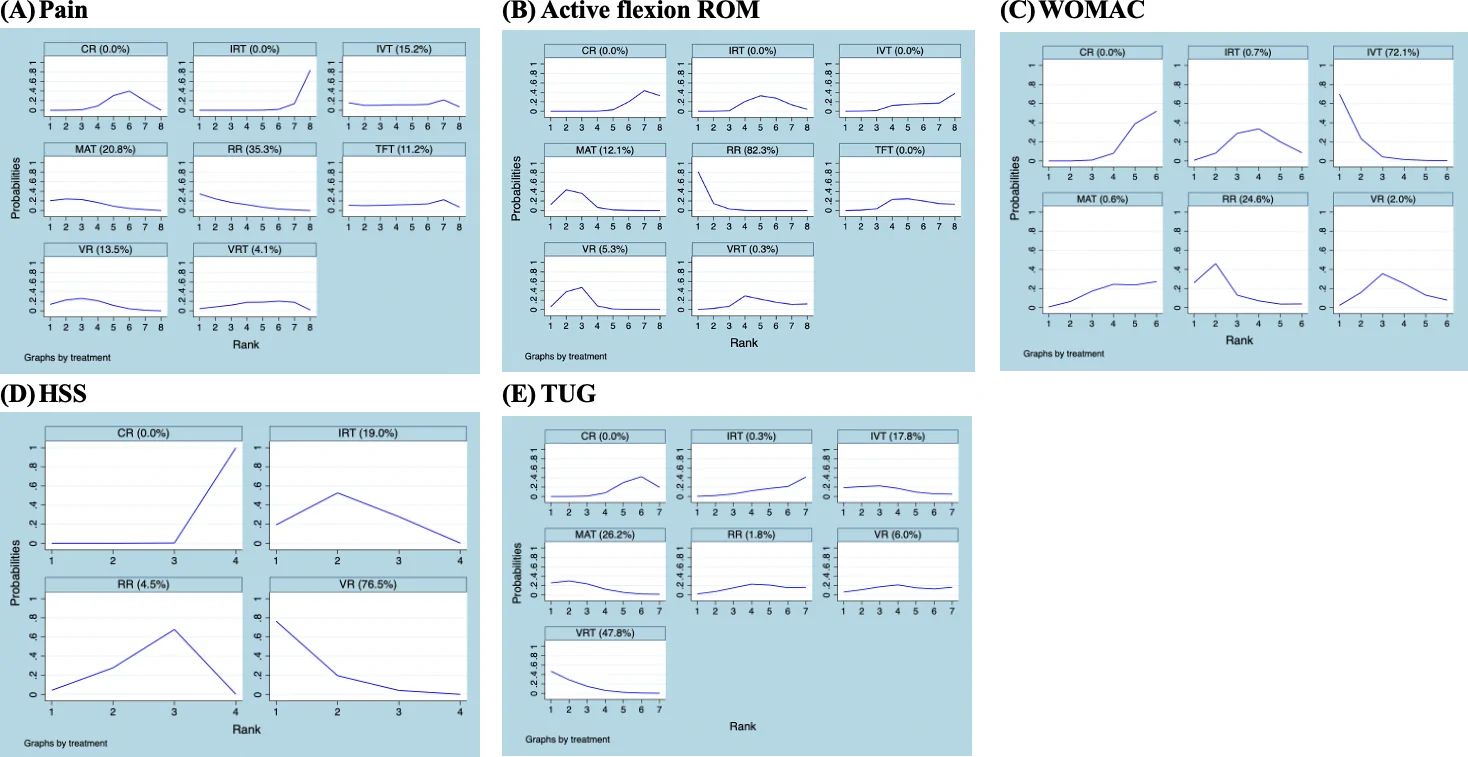
Rankograms. (A) Pain, (B) active flexion range of motion (ROM), (C) Western Ontario and McMaster Universities Osteoarthritis Index (WOMAC), (D) Hospital for Special Surgery (HSS) knee score, and (E) Timed Up and Go (TUG) test. CR: conventional rehabilitation; IRT: intelligent rehabilitation system-based telerehabilitation; IVT: internet videoconference-based telerehabilitation; MAT: mobile app–based telerehabilitation; RR: rehabilitation robots; TFT: telephone follow-up-based telerehabilitation; VR: virtual reality; VRT: VR-based telerehabilitation.

#### Active Flexion ROM

Compared with CR, RR (MD 8.96, 95% CI 5.86-12.05), MAT (MD 6.36, 95% CI 2.81-9.91), and VR (MD 5.92, 95% CI 2.94-8.90) were significantly superior; IRT, IVT, TFT, and VRT showed no significant difference (Figure 3B). Among digital interventions, RR, MAT, and VR were significantly superior to IRT. SUCRA rankings were, in order, RR (97.0%), MAT (79.8%), VR (76.4%), VRT (39.1%), IRT (36.2%), TFT (35.3%), IVT (22.9%), and CR (13.4%) (Figure 4B). RR had the highest probability of ranking first (82.3%) (Figure 5B).

#### WOMAC Scores

Compared with CR, RR (MD 8.96, 95% CI 5.86-12.05), MAT (MD 6.36, 95% CI 2.81-9.91), and VR (MD 5.92, 95% CI 2.94-8.90) were significantly superior; IRT, IVT, TFT, and VRT showed no significant difference (Figure 3B). Among digital interventions, RR, MAT, and VR were significantly superior to IRT. SUCRA rankings were, in order, RR (97.0%), MAT (79.8%), VR (76.4%), VRT (39.1%), IRT (36.2%), TFT (35.3%), IVT (22.9%), and CR (13.4%) (Figure 4B). RR had the highest probability of ranking first (82.3%) (Figure 5B).

#### HSS Scores

Compared with CR, VR (MD 9.53, 95% CI 5.18-13.88), IRT (MD 6.90, 95% CI 2.35-11.44), and RR (MD 5.32, 95% CI 1.61-9.04) were significantly superior (Figure 3D). No comparisons among digital interventions were statistically significant. SUCRA rankings were, in order, VR (90.8%), IRT (63.2%), RR (45.9%), and CR (0.1%; Figure 4D). VR had the highest probability of ranking first (76.5%; Figure 5D).

#### TUG

Compared with CR, VRT (MD −4.80, 95% CI −8.65 to 0.95) was significantly superior (Figure 3E). No comparisons among digital interventions were statistically significant. SUCRA rankings were, in order, VRT (85.1%), MAT (74.7%), IVT (64.8%), VR (44.7%), RR (38.6%), CR (21.8%), and IRT (20.3%; Figure 4E). VRT had the highest probability of ranking first (47.8%; Figure 5E).

### Heterogeneity and PrIs

Pain scores: *τ²*=0.2642; *I²*=73.4%; 95% PrI –1.38 to 0.83; Active Flexion ROM: *τ²*=13.1086; *I²*=83.2%; 95% PrI –3.74 to 11.68; WOMAC scores: τ²=11.2985; *I²*=87.1%; 95% PrI –10.56 to 5.01; HSS scores: *τ²*=7.7743; *I²*=83.1%; 95% PrI –0.94 to 12.69; TUG: *τ²*=2.4822; *I²*=87.3%; 95% PrI –5.75 to 1.60 (Multimedia Appendix 3). The 95% PrI for each comparison is shown in Figure 3. PrIs were generally wider than CIs, and some crossed the line of no effect, suggesting that future studies may show effects in different directions after considering heterogeneity.

### Subgroup Analysis

By intervention type, VR, RR, and MAT showed statistically significant effects for pain (MD –0.55, –0.69, –0.56; *P*=.21); for ROM, VR, IRT, RR, and MAT were significant (MD 5.55, 1.71, 8.38, 7.42; *P*=.005); for WOMAC, IVT, VR, and RR were significant (MD –1.10, –3.20, –7.21; *P*<.001); for HSS, VR, IRT, and RR were significant (MD 9.63, 2.82, 5.16; *P*<.001); for TUG, only IVT and RR were significant (MD –2.72, –0.94; *P*=.42). Within the IRT node, the wearable sensor subgroup was significant for ROM (MD 2.80, 95% CI 1.19-4.41) and HSS (MD 2.82, 95% CI 0.99-4.65), while the camera and web platform subgroups were mostly nonsignificant; the between-subgroup difference was significant only for ROM (*P*=.03). By dose matching, in the dose-matched group, most effects were close to null or small; the higher-dose group showed more obvious improvements in ROM (MD 5.38), HSS (MD 5.88), and TUG (MD −1.51); between-subgroup differences were statistically significant for pain (*P*=.009) and ROM (*P*=.003). By assessment time window, short-term showed a relatively obvious improvement trend, followed by long-term (ROM: MD 2.24, 95% CI 1.00-3.48; HSS: MD 2.96, 95% CI 1.38-4.53), while medium-term had wider CIs; between-subgroup differences were statistically significant for ROM (*P*) and HSS (*P*=.002). By publication language: effects were basically consistent between Chinese- and English-language literature (all *P*>.05), suggesting that language may not be a major source of heterogeneity. These subgroup analyses were exploratory, and residual heterogeneity remained. Details are provided in Multimedia Appendix 4.

### Publication Bias

Because this network was a star-shaped network and had no closed loops and studies were distributed across multiple treatment nodes, comparison-adjusted funnel plots and Egger test had limited power in assessing publication bias. Egger test is particularly susceptible to a small number of studies and large between-comparison heterogeneity; therefore, its results were used only as descriptive information and cannot alone serve as a basis for determining the presence or absence of publication bias. For pain scores, active flexion ROM, WOMAC, HSS, and TUG, comparison-adjusted funnel plots showed that study points were basically symmetrically distributed around the vertical line, with only a few outliers (Figure 6); Egger test did not detect clear statistical evidence of small-study effects (all outcomes *P*>.05; Multimedia Appendix 5). Therefore, this study did not detect clear statistical evidence of small-study effects, but due to limitations in network structure and test power, it cannot be concluded that publication bias does not exist.

**Figure 6.**
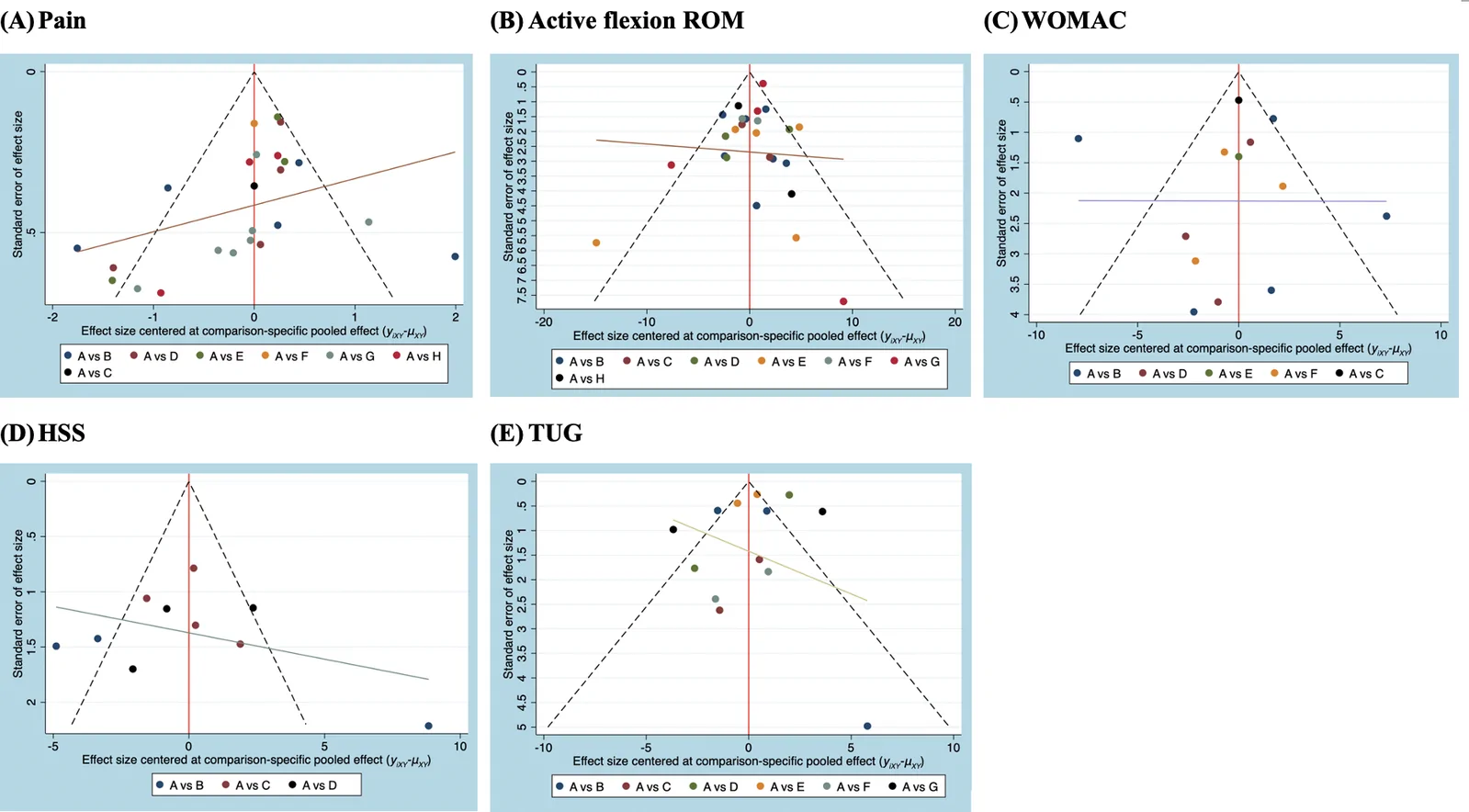
Comparison-adjusted funnel plots. (A) Pain, (B) active flexion range of motion (ROM), (C) Western Ontario and McMaster Universities Osteoarthritis Index (WOMAC), (D) Hospital for Special Surgery (HSS) knee score, and (E) Timed Up and Go (TUG) test.

### Sensitivity Analysis

After leave-one-out analysis, all effect sizes remained within the 95% CI of the overall estimate. After alternative node classification (IDT, TR, RR, CR), the direction and magnitude of effects were basically consistent with the main analysis; for ROM and HSS, IDT, TR, and RR were still significantly superior to CR; for TUG, only IDT was significantly superior to CR; SUCRA rankings were also basically consistent. After excluding high-risk studies, unpublished or non-peer-reviewed sources, very small sample trials, and studies directly reporting SD of change scores, and after using final values instead of change scores, effect directions for all outcomes were basically consistent with the main analysis (mostly *I²*>70%). Overall, the results were robust, but heterogeneity remained high. Because all SDs of change scores were estimated based on *r*=0.5, no further sensitivity analysis using alternative correlation coefficient assumptions was performed. Details of the sensitivity analyses are provided in Multimedia Appendix 6.

### Transitivity Assumption Assessment

Because the network was star-shaped, node-splitting or global inconsistency tests could not be performed; therefore, transitivity was assessed by comparing the distribution of potential effect modifiers among nodes (see box plots in Multimedia Appendix 7). Median age across nodes was generally 65‐70 years, but the age distributions were wider for VR/VRT and RR in several outcomes. Intervention durations differed markedly across nodes and outcomes: TFT had the longest duration for pain and ROM, whereas MAT or IRT had the longest durations for WOMAC, HSS, and TUG; VR and RR generally had shorter durations. VRT and IRT tended to have larger sample sizes, whereas VR, RR, and MAT had smaller sample sizes for most outcomes. Baseline pain, ROM, WOMAC, HSS, and TUG values also showed distribution differences among VR, RR, IRT, and VRT. Unilateral versus bilateral TKA, postoperative intervention start time, rehabilitation setting, type and intensity of CR, risk of bias, and geographic region were compared descriptively and differed across nodes. Overall, the transitivity assumption may not fully hold, and indirect comparisons and SUCRA rankings should be interpreted with caution.

### Certainty of Evidence (CINeMA)

The CINeMA framework was used to assess the certainty of the evidence for the main comparisons and outcomes (Multimedia Appendix 8). Because the network was star-shaped with no closed loops, the incoherence domain was marked as “not assessable” and treated as “major concerns” according to the rules. The certainty of the evidence for most comparisons was low to very low: for pain, CR versus IVT was low and all others very low; for ROM, CR versus MAT was high, CR versus RR and CR versus VR were moderate, CR versus IVT and CR versus VRT were low, and all others very low; for WOMAC, CR versus IVT and CR versus MAT were low and all others very low; for HSS, CR versus VR was moderate, CR versus RR was low, and all others very low; for TUG, all were very low. Direct comparisons (especially CR vs RR, CR vs VR, and CR vs MAT) had relatively higher certainty, while indirect comparisons among digital interventions were all very low, mainly downgraded because of imprecision, heterogeneity, and incoherence. The conclusions should be regarded as hypothesis-generating rather than as definitive evidence.

## Discussion

### Principal Findings

The treatment comparisons in this study involved multiple modes. Under the supplementation mode, the observed benefits may partly come from increased treatment time, supervision, attention, or exercise dose, rather than from the digital health technology itself. We performed subgroup analysis according to whether treatment doses matched. The results showed that in the dose-matched group, pooled effect sizes for most outcomes were close to null or small, while the group in which the experimental group received a higher treatment dose showed more obvious improvement trends in ROM, HSS, and TUG. This suggests that additional treatment dose may be an important factor affecting some outcomes, and the independent effect of digital health technology itself still needs further verification under dose-matched conditions.

### Effects of Digital Health Interventions on Pain in Patients After TKA

This study showed that, compared with CR, RR had the highest SUCRA ranking for pain scores. This is consistent with existing mechanistic and clinical evidence that robot-assisted rehabilitation can reduce joint load through passive/active assistance and promote progressive loading training, thereby relieving pain [10].

The mechanism by which exoskeletons improve pain in patients after TKA is mainly based on their active-assisted movement characteristics. By detecting patients’ muscle bioelectrical signals, exoskeletons can provide precise joint torque support, allowing movements such as knee flexion to be completed without excessive effort by patients, thereby reducing pain stimuli caused by active exertion or improper movement [63,64]. At the same time, this assisted activity helps increase joint ROM, reduce soft tissue adhesions, and promote recovery of muscle strength, indirectly relieving pain and improving joint function [65].

Second, MAT had a relatively high SUCRA ranking for pain relief. One study [66] using a 12-week intelligent KOA lifestyle app intervention found that participants’ knee pain was significantly reduced, and its mechanism was mainly based on 3 aspects: personalized physical activity guidance, educational support, and social interaction. A meta-analysis showed that mobile app–based digital health interventions may have a positive effect on improving pain in patients with KOA [67]. Through structured exercise plans, instant educational content, progress tracking, and social support provided by mobile apps, patients can perform rehabilitation exercises more continuously and accurately, thereby reducing pain, improving knee function, and enhancing quality of life [68].

The finding that CR may be more advantageous than MAT in improving pain should be interpreted with caution because multiple RCTs and systematic reviews have shown that the efficacy of the 2 in pain improvement is often comparable or not significantly different. First, an RCT in patients with KOA compared telerehabilitation with conventional face-to-face rehabilitation. The results showed that both groups had significant improvements in pain scores, but no difference was reported showing that CR was clearly superior to telerehabilitation, suggesting that telerehabilitation is similarly effective rather than clearly inferior to traditional rehabilitation [69]. Second, a systematic review and meta-analysis including multiple RCTs showed that, compared with conventional treatment, telerehabilitation demonstrated a statistically significant improvement in relieving pain in patients with KOA, but the magnitude of improvement was often not much different from face-to-face rehabilitation, indicating that telerehabilitation is not inferior to CR in pain relief [70]. Furthermore, review evidence indicated that telerehabilitation and traditional face-to-face rehabilitation have no significant difference in pain control after knee replacement surgery, further supporting that the 2 are comparable in pain improvement, rather than CR necessarily being superior [71].

However, due to problems such as high dropout rates, intervention heterogeneity, and low evidence quality in existing studies, these findings should be interpreted with caution, and more high-quality studies are still needed for verification in the future.

### Effects of Digital Health Interventions on Active Flexion ROM in Patients After TKA

RR also showed significant advantages in improving active knee flexion ROM, with a relative effect estimate versus CR of MD 8.96 (95% CI 5.86-12.05), and it had the highest SUCRA ranking (97.0%). One study showed that robot-assisted rehabilitation training can significantly improve active knee flexion ROM in older adult patients after TKA. After intervention, the active ROM in the robot group increased from 65.18° to 103.53°, and the passive ROM increased from 82.63° to 104.75°, both significantly superior to the traditional treatment group [32]. This is attributed to the ability of robotic systems to provide high-intensity, repetitive, and consistent training and to combine passive and active movement at different rehabilitation stages through human-machine interaction, thereby more effectively promoting joint function recovery [24,72]. In addition, multiple included studies and meta-analyses showed that robot-assisted rehabilitation has positive effects on improving ROM in patients after total knee or hip arthroplasty [10]. Robot training provides high-intensity, repetitive, and physiologically patterned movements, helping patients complete a larger ROM with less pain, thereby effectively promoting joint function recovery [10].

Second, this study showed that VR ranked next in SUCRA for active flexion ROM. A meta-analysis showed that VR-assisted rehabilitation can more significantly improve knee ROM in patients after TKA compared with traditional rehabilitation [73]. This improvement is mainly attributed to the immersive and interactive training environment provided by VR technology [74,75]. It can not only improve patient participation and exercise adherence through visual feedback and gamified design [23], but also guide patients to complete larger-range and more precise joint movement exercises in a safe and controllable virtual scene, thereby effectively promoting postoperative joint function recovery [20,26].

### Effects of Digital Health Interventions on Knee Function in Patients After TKA

This study showed that, compared with CR, none of the included digital health technologies significantly reduced WOMAC scores in patients after TKA. However, traditional meta-analysis results showed that after pooling all digital health technologies, the improvement effect was significant. Subgroup analysis showed that VR significantly reduced WOMAC scores, which is consistent with Zhai et al [13], especially with a more obvious effect in the hospital setting, possibly related to real-time professional guidance and higher patient adherence [23].

For HSS scores, VR had the highest SUCRA ranking. A meta-analysis found that VR rehabilitation significantly improved HSS scores in patients after TKA in both the early postoperative period (within 1 month) and the medium- to long-term period (2‐3 months), indicating a positive effect on knee function recovery [76,77]. VR rehabilitation can improve patient rehabilitation participation and training adherence through its immersive and interactive environment, thereby improving knee function and showing significant advantages in HSS scores. Specifically, the real-time visual feedback and task-oriented training provided by VR technology may promote neuromuscular control and motor learning processes, helping knee function recovery [76,77]. As an active and interesting rehabilitation mode, VR technology may bring additional functional benefits on top of CR by enhancing patient motivation and distracting attention from pain.

For the TUG test, MAT had the highest SUCRA ranking. One study showed that after using a mobile app (RateFastGonio) to assess knee joint position sense, TUG test times in patients with KOA and after TKA were significantly longer than those in healthy controls, indicating impaired mobility and dynamic balance [78]. The mechanism lies in the fact that TUG is a functional test that comprehensively reflects lower limb muscle strength, balance, coordination, and movement speed [79]; patients with KOA have limited activity due to joint pain, stiffness, and kinesiophobia, leading to decreased muscle strength and abnormal gait, thereby prolonging TUG time [78]; although TKA aims to relieve pain and restore joint alignment, intraoperative removal of intra-articular structures (such as the meniscus and cruciate ligaments) containing mechanoreceptors that provide proprioceptive input may lead to incomplete postoperative proprioceptive recovery [80]. In addition, possible postoperative quadriceps weakness, altered neuromuscular control, and persistent kinesiophobia [81] jointly affect the smoothness and speed of movements such as standing up, walking, and turning, thus manifesting as prolonged TUG time [78].

VRT ranked next in improving TUG scores. Immersive virtual environments provide enhanced multisensory feedback and awareness of results, helping patients better perceive and optimize movement quality, thereby improving motor control and coordination [82,83]. The high interactivity and gamified elements of VR training significantly enhance patient participation and training motivation, which may encourage patients to perform more extensive and higher-intensity functional training, benefiting improvement in daily activity ability [84,85]. Repetitive task-oriented training combined with sensory stimulation helps promote brain plasticity, integrate perception, cognition, and action, thereby consolidating learning effects and enhancing patients’ self-efficacy, laying a neural foundation for improvement in functional activities [82,83]. The remote home training mode overcomes travel barriers, ensuring continuity and accessibility of rehabilitation training and providing conditions for continuous functional improvement [86]. These factors work together and ultimately manifest as improvements in functional activity tests such as TUG.

### Interpretation of SUCRA Rankings

The highest SUCRA ranking does not equate to statistically supported superiority. This network was mainly star-shaped, with no closed loops and few direct comparisons; therefore, comparisons among interventions mainly relied on indirect comparisons through CR; in addition, the certainty of evidence for most comparisons was low to very low.

Residual heterogeneity remained high for some outcomes (*I*²>75%), and prediction intervals were wide, and some crossed the line of no effect, further reducing the certainty of SUCRA ranking results. Therefore, SUCRA rankings should be interpreted as ranking probabilities rather than definitive evidence of treatment superiority [87,88]. When referring to ranking results, clinicians should comprehensively judge based on relative effect estimates, certainty of evidence, residual heterogeneity, and MCID, rather than relying solely on SUCRA rankings.

### Consideration of Treatment Comparisons and Additional Treatment Exposure

The treatment comparisons in this study were not a single mode. According to the relationship between digital health interventions and CR, included studies could be divided into 4 modes: digital health intervention replacing CR, digital health intervention supplementing CR, CR delivered digitally, and self-directed versus clinician-supervised comparison. Under the supplementary mode, the experimental group received “CR+digital health intervention,” while the control group received only CR. Therefore, the observed benefits may come not only from the digital health technology itself but also partly from increased treatment time, supervision, attention, or exercise dose. Existing studies have shown that in rehabilitation after TKA, additional device-assisted training [89] or higher-intensity strength training [90] can bring functional gains superior to standard rehabilitation, suggesting that treatment dose and training intensity themselves are important factors affecting outcomes. Another systematic review also pointed out that the content and dose of postoperative rehabilitation programs vary greatly and are related to outcome improvement [91].

We performed subgroup analysis according to whether treatment doses matched. The results showed that in the dose-matched group, pooled effect sizes for most outcomes were close to null or small, while the group with higher dose in the experimental group showed more obvious improvement trends in active flexion ROM, HSS, and TUG. In addition, the CR node itself was relatively broad, including forms such as physical therapy, routine care, balance training, continuous passive motion, and health education, and its internal heterogeneity may affect the reliability of indirect comparisons. This heterogeneity has been partly considered in the transitivity assumption assessment of the NMA by comparing the distribution of effect modifiers among nodes. Therefore, the independent effect of digital health technology itself still needs further verification under dose-matched conditions.

### Distinguishing Statistical Improvement From Clinically Meaningful Improvement

Statistical significance does not equate to clinical significance; even if an intervention reaches statistical significance, its effect size may not exceed the MCID, and vice versa. Therefore, while reporting statistical significance, this study further assessed clinical significance with reference to MCID. For pain, the MCID using VAS 0‐100 mm was 15 mm [58], which converts to approximately 2 points on a 0‐10 scale [58]; the MCID for ROM was 9° [59]; the MCID for WOMAC total score was 15 points [60]; the MCID for HSS was 5.41 points [61]; the MCID for TUG was 1.4 seconds [62].

The results showed that the lower limits of the 95% CI for HSS (VR vs CR, MD 9.53, 95% CI 5.18, 13.88) and TUG (VRT vs CR, MD –4.80, 95% CI –8.65 to –0.95) both exceeded the corresponding MCID (HSS 5.41 points [61]; TUG 1.4 s [62]), suggesting that these improvements are not only statistically significant but may also be clinically meaningful. In contrast, the lower limits of the 95% CI for pain (RR vs CR, MD −0.81, 95% CI –1.73 to 0.11), ROM (RR vs CR, MD 8.96, 95% CI 5.86-12.05), and WOMAC (IVT vs CR, MD –11.00, 95% CI –19.03 to –2.97) did not exceed the MCID (pain 2 points [58]; ROM 9° [59]; WOMAC 15 points [60]), suggesting that their clinical significance remains unclear. It should be noted that the MCID for TUG came from a hip osteoarthritis population and may not be fully applicable to the TKA population; caution is needed in interpretation.

The above results indicate that the statistical advantages of some interventions may translate into clinical benefits, but performance differs across outcomes; when referring to ranking results, clinicians should comprehensively judge in combination with MCID, rather than relying solely on statistical significance or SUCRA rankings. Future studies should report more clinical significance analyses using MCID as a threshold to support individualized rehabilitation decisions.

### Equity and Accessibility in Digital Rehabilitation

The interventions included in this study involved home telerehabilitation, mobile apps, videoconferencing, wearable technology, and intelligent rehabilitation systems. Although these methods can improve rehabilitation accessibility, they may also raise important equity, privacy, and data security issues. First, digital accessibility and affordability may affect whether patients can participate in telerehabilitation; patients lacking smartphones, tablets, or stable internet services may be excluded [92]. Second, differences in digital literacy may affect older patients’ ability to use technology, thereby affecting intervention adoption and adherence [93]. Third, privacy protection, informed consent, and data security are issues that must be considered in telerehabilitation, especially when using wearable devices and web platforms [94]. Fourth, people with disabilities, linguistic minority groups, and residents in remote areas may face additional accessibility barriers [95]. Fifth, the availability of caregivers or technical support may affect the implementation effect of interventions [96]. These factors may not only affect intervention adoption and adherence but may also affect their effectiveness, and therefore caution is needed when interpreting the results of this study.

In addition, among the included studies, only a few reported device or internet accessibility, digital literacy, or technical support; most studies did not assess socioeconomic characteristics, usability, accessibility, privacy, or privacy- and equity-related outcomes [97]. Future studies should systematically evaluate these equity and privacy issues when designing and reporting telerehabilitation interventions.

### Limitations

This study has the following limitations. There was considerable variation among studies in intervention duration, protocol, and device type; subtypes within each category of digital health technology were insufficiently differentiated; the CR node was relatively broad (including physical therapy, routine care, balance training, continuous passive motion, health education, etc); and the IRT node contained different technology combinations such as wearable sensors, cameras, and web platforms. In addition, the intervention duration and assessment time points of included studies varied greatly, with the main analysis still pooling data from different time points. All these factors may introduce heterogeneity and affect the reliability of indirect comparisons and ranking results.

Most existing studies compared digital health technology with CR, lacking direct comparisons among different digital health technologies; some technologies (such as AR and telephone follow-up telerehabilitation) had relatively small sample sizes; and this network was mainly a star-shaped network with no closed loops, making node-splitting or global inconsistency tests impossible. These factors together limit the credibility and comparative stability of the NMA. Future studies should encourage multi-arm direct comparison trials and conduct more high-quality research.

The transitivity assessment showed that some effect modifiers (such as intervention duration, baseline active flexion ROM, baseline TUG, baseline HSS, and sample size) differed markedly in distribution among treatment nodes, suggesting that the transitivity assumption may not fully hold. Factors such as unilateral versus bilateral TKA, postoperative intervention start time, rehabilitation setting, type and intensity of CR, risk of bias, and geographic region were only descriptively compared and not quantitatively analyzed, so their impact could not be fully assessed.

Regarding statistical methods, this study used *r*=0.5 to estimate the SD of change scores and did not perform sensitivity analysis with alternative correlation coefficient assumptions; *τ²* for each outcome was estimated using the DL method; some outcomes had high heterogeneity (*I²*>75%), and prediction intervals were wide and crossed the line of no effect; and most included studies did not directly report the SD of change scores, so formula-based estimation may affect study weights, SE, and heterogeneity estimates. Because only 2 studies directly reported this parameter, the impact of imputed SD could not be fully assessed, and results should be interpreted with caution.

In addition, the MCID for TUG came from a hip osteoarthritis population and may not be fully applicable to the TKA population; a TUG MCID specific to the post-TKA population should be established in the future. The study also did not perform detailed stratification of the patient population (such as by age or preoperative function), making it difficult to achieve individualized efficacy assessment; future studies should strengthen subgroup analyses to support the development of precision rehabilitation programs.

### Conclusion

This study used NMA to evaluate the effects of different digital health interventions on rehabilitation outcomes in patients after TKA. The results showed that, compared with CR, RR, and VR, MAT had relatively high-ranking probabilities for improving postoperative pain, joint ROM, and knee function. Based on the available evidence, the effects of VR in improving HSS and VRT in improving TUG may be clinically meaningful, while the clinical significance of pain scores, active flexion ROM, and WOMAC remains unclear.

However, because this network was mainly a star-shaped network with no closed loops, sparse direct comparisons, high heterogeneity in intervention protocols, some studies at high risk of bias, low to very low certainty of evidence for most comparisons, and residual high heterogeneity for some outcomes, the above rankings should be interpreted as ranking probabilities rather than definitive evidence of comparative superiority. Therefore, the relative superiority among different digital health interventions remains uncertain, and the above conclusions still need to be verified by more high-quality, head-to-head RCTs, ultimately to develop efficient and personalized rehabilitation programs for patients after TKA.

## Supplementary material

10.2196/101277 Multimedia Appendix 1 Search strategy.

10.2196/101277 Multimedia Appendix 2 Risk of bias.

10.2196/101277 Multimedia Appendix 3 Prediction interval.

10.2196/101277 Multimedia Appendix 4 Subgroup analysis.

10.2196/101277 Multimedia Appendix 5 Egger test.

10.2196/101277 Multimedia Appendix 6 Sensitivity analyses.

10.2196/101277 Multimedia Appendix 7 Transitivity assumption.

10.2196/101277 Multimedia Appendix 8 CINeMA (confidence in network meta-analysis) assessment.

10.2196/101277 Checklist 1 PRISMA network meta-analysis checklist.

## Abbreviations

AR: augmented reality
CINeMA: confidence in network meta-analysis
CR: conventional rehabilitation
HSS: Hospital for Special Surgery
IRT: intelligent rehabilitation system-based telerehabilitation
ITT: intention-to-treat
IVT: internet videoconference-based telerehabilitation
KOA: knee osteoarthritis
MAT: mobile app–based telerehabilitation
MCID: minimal clinically important difference
MD: mean difference
NMA: network meta-analysis
NPRS: numeric pain rating scale
OKS: Oxford Knee Score
PrI: prediction interval
PRISMA: Preferred Reporting Items for Systematic Reviews and Meta-Analyses
PROSPERO: International Prospective Register of Systematic Reviews
RCT: randomized controlled trial
RoB 2: Risk of Bias 2
ROM: range of motion
RR: rehabilitation robot
SMD: standardized mean difference
SUCRA: surface under the cumulative ranking curve
TFT: telephone follow-up-based telerehabilitation
TKA: total knee arthroplasty
TUG: Timed Up and Go
VAS: visual analog scale
VR: virtual reality
VRT: virtual reality–based telerehabilitation
WOMAC: Western Ontario and McMaster Universities Osteoarthritis Index
