# Supplementary material for: Effectiveness of Digital Health Interventions on Rehabilitation Outcomes in Patients After Total Knee Arthroplasty: Systematic Review and Network Meta-Analysis
Source: J Med Internet Res. 2026 Nov 13;28:e101277. doi: 10.2196/101277 (PMC13762687; doi:10.2196/101277)
Supplement: Supplementary file 1 [file jmir-v28-e101277-s001.docx]

**Multimedia Appendix 2. Search strategy.**

**Table S2.** Database search strategy.

| **Database** | Search strategies |
| --- | --- |
| **PubMed** | [#1] "Arthroplasty, Replacement, Knee"[MeSH] OR "Knee Replacement, Total"[MeSH] OR total knee replacement[Title/Abstract] OR TKR[Title/Abstract] OR total knee arthroplasty[Title/Abstract] OR TKA[Title/Abstract] OR knee prosthesis[Title/Abstract] OR knee replacement surgery[Title/Abstract]  [#2] "Artificial Intelligence"[MeSH] OR "Virtual Reality"[MeSH] OR "Wearable Electronic Devices"[MeSH] OR "Telemedicine"[MeSH] OR "Robotics"[MeSH] OR "Mobile Applications"[MeSH] OR Artificial Intelligence[Title/Abstract] OR AI[Title/Abstract] OR virtual reality[Title/Abstract] OR VR[Title/Abstract] OR wearable devices[Title/Abstract] OR wearable sensor[Title/Abstract] OR telemedicine[Title/Abstract] OR telerehabilitation[Title/Abstract] OR rehabilitation robot[Title/Abstract] OR robot-assisted training[Title/Abstract] OR intelligent rehabilitation system[Title/Abstract] OR mobile application*[Title/Abstract] OR app[Title/Abstract] OR internet video conference[Title/Abstract] OR telephone follow-up[Title/Abstract] OR eHealth[Title/Abstract] OR mHealth[Title/Abstract] OR digital health[Title/Abstract] OR deep learning[Title/Abstract] OR machine learning[Title/Abstract] OR exergame[Title/Abstract] OR exergaming[Title/Abstract] OR serious game[Title/Abstract] OR interactive gaming[Title/Abstract]  [#3] "Randomized Controlled Trial"[Publication Type] OR "Controlled Clinical Trial"[Publication Type] OR randomized controlled trial[Title/Abstract] OR RCT[Title/Abstract] OR controlled clinical trial[Title/Abstract] OR clinical trial[Title/Abstract]  [#4] [#1] AND [#2] AND [#3] |
| **Embase** | [#1] 'knee replacement'/exp OR 'total knee arthroplasty'/exp OR 'total knee replacement':ab,ti,kw OR 'tkr':ab,ti,kw OR 'total knee arthroplasty':ab,ti,kw OR 'tka':ab,ti,kw OR 'knee prosthesis':ab,ti,kw  [#2] 'artificial intelligence'/exp OR 'virtual reality'/exp OR 'wearable device'/exp OR 'telemedicine'/exp OR 'telerehabilitation'/exp OR 'rehabilitation robot'/exp OR 'mobile application'/exp OR 'ai':ab,ti,kw OR 'virtual reality':ab,ti,kw OR 'vr':ab,ti,kw OR 'wearable device':ab,ti,kw OR 'wearable sensor':ab,ti,kw OR 'telemedicine':ab,ti,kw OR 'telerehabilitation':ab,ti,kw OR 'rehabilitation robot':ab,ti,kw OR 'robot-assisted training':ab,ti,kw OR 'intelligent rehabilitation system':ab,ti,kw OR 'mobile app':ab,ti,kw OR 'ehealth':ab,ti,kw OR 'mhealth':ab,ti,kw OR 'digital health':ab,ti,kw OR 'deep learning':ab,ti,kw OR 'machine learning':ab,ti,kw OR 'telephone follow-up':ab,ti,kw OR 'internet video conference':ab,ti,kw OR 'exergame':ab,ti,kw OR 'exergaming':ab,ti,kw OR 'serious game':ab,ti,kw OR 'interactive gaming':ab,ti,kw  [#3] 'randomized controlled trial'/exp OR 'rct':ab,ti,kw OR 'controlled clinical trial':ab,ti,kw  [#4] [#1] AND [#2] AND [#3] |
| **Cochrane library** | [#1] MeSH descriptor: [Arthroplasty, Replacement, Knee] explode all trees OR (total knee replacement or TKR or total knee arthroplasty or TKA or knee prosthesis):ti,ab,kw  [#2] MeSH descriptor: [Artificial Intelligence] explode all trees OR MeSH descriptor: [Virtual Reality] explode all trees OR MeSH descriptor: [Wearable Electronic Devices] explode all trees OR MeSH descriptor: [Telemedicine] explode all trees OR MeSH descriptor: [Robotics] explode all trees OR (Artificial Intelligence or AI or virtual reality or VR or wearable devices or wearable sensors or telemedicine or telerehabilitation or rehabilitation robot* or robot-assisted training or intelligent rehabilitation system or mobile application* or app or eHealth or mHealth or digital health or deep learning or machine learning or telephone follow-up or internet video conference or exergame or exergaming or serious game or interactive gaming):ti,ab,kw  [#3] MeSH descriptor: [Randomized Controlled Trial] explode all trees OR (randomized controlled trial or RCT or controlled clinical trial):ti,ab,kw  [#4] [#1] AND [#2] AND [#3] |
| **Web of Science** | [#1] TS=(total knee replacement OR TKR OR total knee arthroplasty OR TKA OR knee prosthesis)  [#2] TS=(artificial intelligence OR AI OR virtual reality OR VR OR wearable devices OR wearable sensors OR telemedicine OR telerehabilitation OR rehabilitation robot* OR robot-assisted training OR intelligent rehabilitation system OR mobile application* OR app OR eHealth OR mHealth OR digital health OR deep learning OR machine learning OR telephone follow-up OR internet video conference OR exergame OR exergaming OR serious game OR interactive gaming)  [#3] TS=(randomized controlled trial OR RCT OR controlled clinical trial)  [4#] [#1] AND [#2] AND [#3] |
| **Scopus** | [#1] TITLE-ABS-KEY("total knee replacement" OR "tkr" OR "total knee arthroplasty" OR "tka" OR "knee prosthesis")  [#2] TITLE-ABS-KEY("artificial intelligence" OR "ai" OR "virtual reality" OR "vr" OR "wearable devices" OR "wearable sensors" OR "telemedicine" OR "telerehabilitation" OR "rehabilitation robot" OR "robot-assisted training" OR "intelligent rehabilitation system" OR "mobile application" OR "app" OR "ehealth" OR "mhealth" OR "digital health" OR "deep learning" OR "machine learning" OR "telephone follow-up" OR "internet video conference" OR "exergame" OR "exergaming" OR "serious game" OR "interactive gaming")  [#3] TITLE-ABS-KEY("randomized controlled trial" OR "rct" OR "controlled clinical trial")  [#4] [#1] AND [#2] AND [#3] |
| **Google Scholar** | [#1] "total knee replacement" OR "TKR" OR "total knee arthroplasty" OR "TKA" OR "knee replacement surgery"  [#2] "artificial intelligence" OR "AI" OR "virtual reality" OR "VR" OR "wearable devices" OR "telemedicine" OR "telerehabilitation" OR "rehabilitation robot" OR "mobile application" OR "deep learning" OR "machine learning" OR "exergame" OR "exergaming" OR "serious game" OR "interactive gaming"  [#3] "RCT" OR "randomized controlled trial" OR "randomised controlled trial"  [#4] [#1] AND [#2] AND [#3] |
| **CINAHL** | [#1] MH Arthroplasty, Replacement, Knee OR TI (total knee replacement OR TKR OR total knee arthroplasty OR TKA OR knee prosthesis OR knee replacement surgery OR knee arthroplasty) OR AB (total knee replacement OR TKR OR total knee arthroplasty OR TKA OR knee prosthesis OR knee replacement surgery OR knee arthroplasty)  [#2] MH artificial intelligence OR MH virtual reality OR MH wearable devices OR MH telemedicine OR MH telerehabilitation OR MH robotics OR MH mobile applications OR TI (artificial intelligence OR AI OR virtual reality OR VR OR wearable devices OR wearable sensors OR telemedicine OR telerehabilitation OR rehabilitation robot* OR robot-assisted training OR intelligent rehabilitation system* OR mobile application* OR app OR eHealth OR mHealth OR digital health OR deep learning OR machine learning OR telephone follow-up OR internet video conference OR artificial intelligence-based rehabilitation OR smart healthcare OR digital therapy OR exergame OR exergaming OR serious game OR interactive gaming) OR AB (artificial intelligence OR AI OR virtual reality OR VR OR wearable devices OR wearable sensors OR telemedicine OR telerehabilitation OR rehabilitation robot* OR robot-assisted training OR intelligent rehabilitation system* OR mobile application* OR app OR eHealth OR mHealth OR digital health OR deep learning OR machine learning OR telephone follow-up OR internet video conference OR artificial intelligence-based rehabilitation OR smart healthcare OR digital therapy OR exergame OR exergaming OR serious game OR interactive gaming)  [#3] MH Randomized Controlled Trials OR TI (randomized controlled trial* OR RCT OR clinical trials, randomized OR trials, randomized clinical OR controlled clinical trials, randomized) OR AB (randomized controlled trial* OR RCT OR clinical trials, randomized OR trials, randomized clinical OR controlled clinical trials, randomized)  [#4] [#1] AND [#2] AND [#3] |
| **MEDLINE via OvidSP** | [#1] exp Arthroplasty, Replacement, Knee/ OR (total knee replacement or TKR or total knee arthroplasty or TKA or knee prosthesis or knee replacement surgery or knee arthroplasty).ab,ti,kw.  [#2] exp Artificial Intelligence/ OR exp Virtual Reality/ OR exp Wearable Electronic Devices/ OR exp Telemedicine/ OR exp Telerehabilitation/ OR exp Robotics/ OR exp Mobile Applications/ OR (artificial intelligence or AI or virtual reality or VR or wearable devices or wearable sensors or telemedicine or telerehabilitation or rehabilitation robot* or robot-assisted training or intelligent rehabilitation system* or mobile application* or app or eHealth or mHealth or digital health or deep learning or machine learning or telephone follow-up or internet video conference or artificial intelligence-based rehabilitation or smart healthcare or digital therapy or exergame or exergaming or serious game or interactive gaming).ab,ti,kw.  [#3] exp Randomized Controlled Trial/ OR (randomized controlled trial* or RCT or clinical trials, randomized or trials, randomized clinical or controlled clinical trials, randomized).ab,ti,kw.  [#4] [#1] AND [#2] AND [#3] |
| **China National Knowledge Infrastructure (CNKI)** | [#1] Subject = total knee arthroplasty OR Title/Keyword/Abstract = total knee replacement OR TKR OR TKA OR knee replacement OR artificial knee OR knee arthroplasty  [#2] Subject = artificial intelligence OR Title/Keyword/Abstract = artificial intelligence OR AI OR virtual reality OR VR OR wearable devices OR wearable sensors OR telemedicine OR telerehabilitation OR rehabilitation robot OR robot-assisted training OR intelligent rehabilitation system OR mobile application OR app OR APP OR internet video conference OR telephone follow-up OR eHealth OR mHealth OR digital health OR deep learning OR machine learning OR exergame OR exergaming OR serious game OR interactive gaming  [#3] Subject = randomized controlled trial OR Title/Keyword/Abstract = randomized controlled trial OR RCT OR clinical trial OR controlled trial  [#4] [#1] AND [#2] AND [#3] |
| **Wanfang data** | [#1] Subject = total knee arthroplasty OR Title/Keyword = total knee replacement OR TKR OR TKA OR knee replacement OR artificial knee OR knee arthroplasty OR knee replacement surgery  [#2] Subject = artificial intelligence OR Title/Keyword = artificial intelligence OR AI OR virtual reality OR VR OR wearable devices OR wearable sensors OR telemedicine OR telerehabilitation OR rehabilitation robot OR robot-assisted training OR intelligent rehabilitation system OR mobile application OR app OR APP OR internet video conference OR telephone follow-up OR eHealth OR mHealth OR digital health OR deep learning OR machine learning OR artificial intelligence rehabilitation OR smart healthcare OR digital therapy OR exergame OR exergaming OR serious game OR interactive gaming  [#3] Subject = randomized controlled trial OR Title/Keyword = randomized controlled trial OR RCT OR clinical trial OR controlled trial OR randomized OR random allocation  [#4] [#1] AND [#2] AND [#3] |
| **Chinese Scientific Journal database (VIP)** | [#1] Title/Keyword = total knee arthroplasty OR Title/Keyword = TKR OR Title/Keyword = TKA OR Title/Keyword = knee replacement OR Title/Keyword = artificial knee OR Title/Keyword = knee arthroplasty OR Title/Keyword = knee replacement surgery  [#2] Title/Keyword = artificial intelligence OR Title/Keyword = AI OR Title/Keyword = virtual reality OR Title/Keyword = VR OR Title/Keyword = wearable devices OR Title/Keyword = wearable sensors OR Title/Keyword = telemedicine OR Title/Keyword = telerehabilitation OR Title/Keyword = rehabilitation robot OR Title/Keyword = robot-assisted training OR Title/Keyword = intelligent rehabilitation system OR Title/Keyword = mobile application OR Title/Keyword = app OR Title/Keyword = APP OR Title/Keyword = internet video conference OR Title/Keyword = telephone follow-up OR Title/Keyword = eHealth OR Title/Keyword = mHealth OR Title/Keyword = digital health OR Title/Keyword = deep learning OR Title/Keyword = machine learning OR Title/Keyword = artificial intelligence rehabilitation OR Title/Keyword = smart healthcare OR Title/Keyword = digital therapy OR Title/Keyword = exergame OR Title/Keyword = exergaming OR Title/Keyword = serious game OR Title/Keyword = interactive gaming  [#3] Title/Keyword = randomized controlled trial OR Title/Keyword = RCT OR Title/Keyword = clinical trial OR Title/Keyword = controlled trial OR Title/Keyword = randomized OR Title/Keyword = random allocation  [#4] [#1] AND [#2] AND [#3] |
