# Supplementary material for: Effectiveness of Digital Health Interventions on Rehabilitation Outcomes in Patients After Total Knee Arthroplasty: Systematic Review and Network Meta-Analysis
Source: J Med Internet Res. 2026 Nov 13;28:e101277. doi: 10.2196/101277 (PMC13762687; doi:10.2196/101277)
Supplement: Supplementary file 2 [file jmir-v28-e101277-s002.docx]

**Multimedia Appendix 3. Risk of bias.**

**Table S3.1. Risk of bias assessment of included studies**

| Study | Outcome type | Outcome measures | D1 | D2 | Participant blinding | D3 | D4 | Assessor blinding | D5 | Analysis type | Overall |
| --- | --- | --- | --- | --- | --- | --- | --- | --- | --- | --- | --- |
| Russell 2011[1] | Subjective | VAS, WOMAC | Low | Low | Not feasible | Low | Low | Yes | Low | ITT | Low |
| Russell 2011[1] | Objective | ROM, TUG | Low | Low | Not feasible | Low | Low | Yes | Low | ITT | Low |
| Tousignant 2011[2] | Objective | TUG | Low | Some concerns | Not feasible | Low | Low | Yes | Low | ITT | Some concerns |
| Piqueras [3] | Subjective | VAS | Low | Low | Not feasible | Low | Low | Yes | Low | ITT | Low |
| Piqueras [3] | Objective | ROM, TUG | Low | Low | Not feasible | Low | Low | Yes | Low | ITT | Low |
| Moffet 2015 [4] | Objective | ROM | Low | Low | Not feasible | Low | Low | Yes | Low | ITT | Low |
| Chen 2016 [5] | Subjective | VAS | Some concerns | Some concerns | Not feasible | Some concerns | Some concerns | Not reported | Some concerns | ITT | Some concerns |
| Chen 2016 [5] | Objective | ROM | Some concerns | Some concerns | Not feasible | Some concerns | Low | Not reported | Some concerns | ITT | Some concerns |
| Jin 2018 [6] | Subjective | VAS, WOMAC, HSS | Some concerns | Some concerns | Not feasible | Low | Some concerns | Not reported | Some concerns | Not reported | Some concerns |
| Jin 2018 [6] | Objective | ROM | Some concerns | Some concerns | Not feasible | Low | Low | Not reported | Some concerns | Not reported | Some concerns |
| Kotani 2020[7] | Subjective | VAS | Low | Low | Not feasible | Low | Some concerns | Yes | Low | ITT | Some concerns |
| Kotani 2020[7] | Objective | ROM | Low | Low | Not feasible | Low | Low | Yes | Low | ITT | Low |
| Prvu Bettger 2020[8] | Subjective | NPRS | Low | Some concerns | Not feasible | Low | Low | Yes | Low | ITT | Some concerns |
| Gianola 2020 [9] | Subjective | VAS, WOMAC | Low | Low | Not feasible | Some concerns | Low | Yes | Low | ITT | Some concerns |
| Gianola 2020 [9] | Objective | ROM | Low | Low | Not feasible | Some concerns | Low | Yes | Low | ITT | Some concerns |
| Yoon 2020[10] | Subjective | WOMAC | Some concerns | Some concerns | Not feasible | Low | Some concerns | Not reported | Some concerns | Not reported | Some concerns |
| Yoon 2020[10] | Objective | TUG | Some concerns | Some concerns | Not feasible | Low | Low | Not reported | Some concerns | Not reported | Some concerns |
| Prabhu 2020[11] | Subjective | VAS | Some concerns | Some concerns | Not feasible | Low | Some concerns | Not reported | Some concerns | Not reported | Some concerns |
| Bäcker 2021[12] | Subjective | VAS | Some concerns | Some concerns | Not feasible | High | Some concerns | Not reported | Some concerns | Not reported | High |
| Pournajaf 2022[13] | Subjective | VAS | Low | Low | Not feasible | Low | Low | Yes | Low | ITT | Low |
| Pournajaf 2022[13] | Objective | TUG | Low | Low | Not feasible | Low | Low | Yes | Low | ITT | Low |
| Mrotzek 2022[14] | Objective | ROM | Low | Low | Not feasible | Low | Some concerns | Yes | Low | ITT | Some concerns |
| Cai 2023[15] | Subjective | HSS | Some concerns | Some concerns | Not feasible | Low | Some concerns | Not reported | Some concerns | Not reported | Some concerns |
| Cai 2023[15] | Objective | ROM | Some concerns | Some concerns | Not feasible | Some concerns | Low | Not reported | Some concerns | Not reported | Some concerns |
| Janhunen 2023[16] | Subjective | VAS | Low | Low | Not feasible | Low | Some concerns | No | Low | ITT | Some concerns |
| Janhunen 2023[16] | Objective | ROM, TUG | Low | Low | Not feasible | Low | Some concerns | No | Low | ITT | Some concerns |
| Shim 2023[17] | Subjective | NPRS, WOMAC | Low | Some concerns | Not feasible | High | Some concerns | Not reported | Low | ITT+PP | High |
| Shim 2023[17] | Objective | ROM | Low | Some concerns | Not feasible | High | Low | Not reported | Low | ITT+PP | High |
| Yu 2023[18] | Subjective | VAS | Low | Low | Not feasible | Low | Low | Yes | Low | ITT | Low |
| Yu 2023[18] | Objective | ROM | Low | Low | Not feasible | Low | Low | Yes | Low | ITT | Low |
| Zhao 2024[19] | Subjective | VAS, WOMAC | Low | Low | Not feasible | Low | Low | Yes | Low | ITT | Low |
| Zhao 2024[19] | Objective | ROM | Low | Low | Not feasible | Low | Low | Yes | Low | ITT | Low |
| Nuevo 2024[20] | Subjective | VAS, WOMAC | Low | Low | Not feasible | Low | Low | Yes | Low | ITT | Low |
| Nuevo 2024[20] | Objective | ROM, TUG | Low | Low | Not feasible | Low | Low | Yes | Low | ITT | Low |
| Pua 2024 [21] | Subjective | NPRS | Low | Low | Not feasible | Low | Low | Yes | Low | ITT | Low |
| Pua 2024 [21] | Objective | ROM | Low | Low | Not feasible | Low | Low | Yes | Low | ITT | Low |
| Bradbury 2024[22] | Subjective | NPRS | Low | Some concerns | Not feasible | Low | Low | Yes | Low | ITT | Some concerns |
| Bradbury 2024[22] | Objective | ROM, TUG | Low | Some concerns | Not feasible | Low | Low | Yes | Low | ITT | Some concerns |
| Pérez-Maletzki 2024[23] | Subjective | VAS, WOMAC | Low | Low | Not feasible | Low | Low | Yes | Low | ITT | Low |
| Pérez-Maletzki 2024[23] | Objective | ROM | Low | Low | Not feasible | Low | Low | Yes | Low | ITT | Low |
| Jin 2017[24] | Subjective | VAS | Some concerns | Some concerns | Not feasible | Low | Some concerns | Not reported | Some concerns | Not reported | Some concerns |
| Sun 2017[25] | Objective | ROM | Some concerns | Some concerns | Not feasible | Some concerns | Low | Not reported | Some concerns | Not reported | Some concerns |
| Li 2018[26] | Subjective | VAS, HSS | Some concerns | Some concerns | Not feasible | Low | Some concerns | Not reported | Some concerns | Not reported | Some concerns |
| Feng 2018[27] | Subjective | WOMAC, HSS | Some concerns | Some concerns | Not feasible | Low | Some concerns | Not reported | Some concerns | Not reported | Some concerns |
| Feng 2018[27] | Objective | ROM | Some concerns | Some concerns | Not feasible | Low | Low | Not reported | Some concerns | Not reported | Some concerns |
| Zhao 2018[28] | Subjective | WOMAC, HSS | Some concerns | Some concerns | Not feasible | Some concerns | Some concerns | Not reported | Some concerns | Not reported | Some concerns |
| Zhao 2018[28] | Objective | ROM | Some concerns | Some concerns | Not feasible | Some concerns | Low | Not reported | Some concerns | Not reported | Some concerns |
| Ding 2019[29] | Subjective | WOMAC, HSS | Some concerns | Some concerns | Not feasible | Some concerns | Some concerns | Not reported | Some concerns | Not reported | Some concerns |
| Ding 2019[29] | Objective | ROM | Some concerns | Some concerns | Not feasible | Some concerns | Low | Not reported | Some concerns | Not reported | Some concerns |
| Zhang 2020[30] | Objective | ROM, TUG | Some concerns | Some concerns | Not feasible | High | Low | Not reported | Some concerns | Not reported | High |
| Li 2021[31] | Subjective | VAS, WOMAC | Some concerns | Some concerns | Not feasible | Low | Some concerns | Not reported | Some concerns | Not reported | Some concerns |
| Li 2021[31] | Objective | TUG | Some concerns | Some concerns | Not feasible | Low | Low | Not reported | Some concerns | Not reported | Some concerns |
| Zhou 2022[32] | Subjective | HSS | Some concerns | Some concerns | Not feasible | Low | Some concerns | Not reported | Some concerns | Not reported | Some concerns |
| Zhou 2022[32] | Objective | ROM | Some concerns | Some concerns | Not feasible | Low | Low | Not reported | Some concerns | Not reported | Some concerns |
| Li YS 2023[33] | Subjective | VAS, WOMAC | Low | Low | Not feasible | Low | Low | Yes | Low | ITT | Low |
| Li YS 2023[33] | Objective | ROM, TUG | Low | Low | Not feasible | Low | Low | Yes | Low | ITT | Low |
| Li 2023[34] | Subjective | VAS, HSS | Some concerns | Some concerns | Not feasible | Low | Low | Not reported | Some concerns | Not reported | Some concerns |
| Li 2023[34] | Objective | ROM | Some concerns | Some concerns | Not feasible | Low | Low | Not reported | Some concerns | Not reported | Some concerns |
| Ma 2023[35] | Subjective | VAS, HSS | Some concerns | Some concerns | Not feasible | Low | Some concerns | Not reported | Some concerns | Not reported | Some concerns |
| Ma 2023[35] | Objective | ROM | Some concerns | Some concerns | Not feasible | Low | Low | Not reported | Some concerns | Not reported | Some concerns |
| Fan 2025[36] | Subjective | VAS, WOMAC, HSS | Some concerns | Some concerns | Not feasible | Low | Some concerns | Not reported | Some concerns | Not reported | Some concerns |
| Fan 2025[36] | Objective | ROM, TUG | Some concerns | Some concerns | Not feasible | Low | Low | Not reported | Some concerns | Not reported | Some concerns |

D1, randomization process; D2, deviations from intended interventions; D3, missing outcome data; D4, measurement of the outcome; D5, selection of the reported result; ITT, Intention-to-treat; VAS, Visual Analogue Scale; NPRS, Numeric Pain Rating Scale; ROM, Active Flexion Range of Motion; TUG, Timed Up and Go test; WOMAC, Western Ontario and McMaster Universities Osteoarthritis Index; HSS, Hospital for Special Surgery Knee Score.

- **D1 High risk**: Random sequence generation or allocation concealment was inadequate, or the randomization method was not reported and selection bias could not be excluded.
- **D2 High risk**: There were evident deviations from the intended interventions, and intention-to-treat (ITT) analysis was not used or only per-protocol (PP) analysis was reported.
- **D3 High risk**: Attrition rate > 20%, or markedly imbalanced missing data between groups, or missing outcome data/attrition and the handling of missing data were not reported.
- **D4 High risk**: Assessor blinding was not reported, the outcome was subjective (e.g., pain, WOMAC, HSS), and the measurement could plausibly be influenced by knowledge of the intervention received.
- **D5 High risk**: No trial registration number was reported and there were signs of selective reporting (e.g., not all prespecified outcomes were reported, or only positive results were reported).

**(A)**

**(B)**

**(C)**

**(D)**

**Figure S3.1.** Risk-of-bias summary for the 36 randomized controlled trials assessed with the Cochrane RoB 2 tool.

A Subjective outcomes: proportion of trials assigned low, some-concern, or high risk for each domain, expressed as percentages.

B Subjective outcomes: individual risk-of-bias judgments for all five domains and overall risk of bias across each included trial.

C Objective outcomes: proportion of trials assigned low, some-concern, or high risk for each domain, expressed as percentages.

D Objective outcomes: individual risk-of-bias judgments for all five domains and overall risk of bias across each included trial.

1.	Russell TG, Buttrum P, Wootton R, Jull GA. Internet-based outpatient telerehabilitation for patients following total knee arthroplasty: a randomized controlled trial. J Bone Joint Surg Am. 2011 Jan 19;93(2):113-20. PMID: 21248209. doi: 10.2106/jbjs.I.01375.

2.	Tousignant M, Moffet H, Boissy P, Corriveau H, Cabana F, Marquis F. A randomized controlled trial of home telerehabilitation for post-knee arthroplasty. J Telemed Telecare. 2011;17(4):195-8. PMID: 21398389. doi: 10.1258/jtt.2010.100602.

3.	Piqueras M, Marco E, Coll M, Escalada F, Ballester A, Cinca C, et al. Effectiveness of an interactive virtual telerehabilitation system in patients after total knee arthoplasty: a randomized controlled trial. J Rehabil Med. 2013 Apr;45(4):392-6. PMID: 23474735. doi: 10.2340/16501977-1119.

4.	Moffet H, Tousignant M, Nadeau S, Mérette C, Boissy P, Corriveau H, et al. In-Home Telerehabilitation Compared with Face-to-Face Rehabilitation After Total Knee Arthroplasty: A Noninferiority Randomized Controlled Trial. J Bone Joint Surg Am. 2015 Jul 15;97(14):1129-41. PMID: 26178888. doi: 10.2106/jbjs.N.01066.

5.	Chen M, Li P, Lin F. Influence of structured telephone follow-up on patient compliance with rehabilitation after total knee arthroplasty. Patient Prefer Adherence. 2016;10:257-64. PMID: 27042020. doi: 10.2147/ppa.S102156.

6.	Jin C, Feng Y, Ni Y, Shan Z. Virtual reality intervention in postoperative rehabilitation after total knee arthroplasty: a prospective and randomized controlled clinical trial. INTERNATIONAL JOURNAL OF CLINICAL AND EXPERIMENTAL MEDICINE. 2018;11(6):6119-24.

7.	Kotani N, Morishita T, Saita K, Kamada S, Maeyama A, Abe H, et al. Feasibility of supplemental robot-assisted knee flexion exercise following total knee arthroplasty. J Back Musculoskelet Rehabil. 2020;33(3):413-21. PMID: 31561326. doi: 10.3233/bmr-181482.

8.	Prvu Bettger J, Green CL, Holmes DN, Chokshi A, Mather RC, 3rd, Hoch BT, et al. Effects of Virtual Exercise Rehabilitation In-Home Therapy Compared with Traditional Care After Total Knee Arthroplasty: VERITAS, a Randomized Controlled Trial. J Bone Joint Surg Am. 2020 Jan 15;102(2):101-9. PMID: 31743238. doi: 10.2106/jbjs.19.00695.

9.	Gianola S, Stucovitz E, Castellini G, Mascali M, Vanni F, Tramacere I, et al. Effects of early virtual reality-based rehabilitation in patients with total knee arthroplasty: A randomized controlled trial. Medicine (Baltimore). 2020 Feb;99(7):e19136. PMID: 32049833. doi: 10.1097/md.0000000000019136.

10.	Yoon S, Son H. Effects of full immersion virtual reality training on balance and knee function in total knee replacement patients: a randomized controlled study. J Mech Med Biol. 2020;20(09):2040007. doi: 10.1142/s0219519420400072.

11.	Prabhu VG, Stanley L, Morgan R. A Biofeedback Enhanced Adaptive Virtual Reality Environment for Managing Surgical Pain and Anxiety. International Journal of Semantic Computing. 2020;14(03):375-93. doi: 10.1142/s1793351x20400152.

12.	Bäcker HC, Wu CH, Schulz MRG, Weber-Spickschen TS, Perka C, Hardt S. App-based rehabilitation program after total knee arthroplasty: a randomized controlled trial. Arch Orthop Trauma Surg. 2021 Sep;141(9):1575-82. PMID: 33547927. doi: 10.1007/s00402-021-03789-0.

13.	Pournajaf S, Goffredo M, Pellicciari L, Piscitelli D, Criscuolo S, Le Pera D, et al. Effect of balance training using virtual reality-based serious games in individuals with total knee replacement: A randomized controlled trial. Ann Phys Rehabil Med. 2022 Nov;65(6):101609. PMID: 34839056. doi: 10.1016/j.rehab.2021.101609.

14.	Mrotzek SJ, Ahmadi S, von Glinski A, Brinkemper A, Aach M, Schildhauer TA, et al. Rehabilitation during early postoperative period following total knee arthroplasty using single-joint hybrid assistive limb as new therapy device: a randomized, controlled clinical pilot study. Arch Orthop Trauma Surg. 2022 Dec;142(12):3941-7. PMID: 34783882. doi: 10.1007/s00402-021-04245-9.

15.	Cai L, Liu Y, Wei Z, Liang H, Liu Y, Cui M. Robot-assisted rehabilitation training improves knee function and daily activity ability in older adults following total knee arthroplasty. Res Nurs Health. 2023 Apr;46(2):203-9. PMID: 36504201. doi: 10.1002/nur.22290.

16.	Janhunen M, Katajapuu N, Paloneva J, Pamilo K, Oksanen A, Keemu H, et al. Effects of a home-based, exergaming intervention on physical function and pain after total knee replacement in older adults: a randomised controlled trial. BMJ Open Sport Exerc Med. 2023;9(1):e001416. PMID: 36896366. doi: 10.1136/bmjsem-2022-001416.

17.	Shim GY, Kim EH, Lee SJ, Chang CB, Lee YS, Lee JI, et al. Postoperative rehabilitation using a digital healthcare system in patients with total knee arthroplasty: a randomized controlled trial. Arch Orthop Trauma Surg. 2023 Oct;143(10):6361-70. PMID: 37129691. doi: 10.1007/s00402-023-04894-y.

18.	Yu JH, Nekar DM, Kang HY, Lee JW, Oh SY. Comparison of Physical Activity Training Using Augmented Reality and Conventional Therapy on Physical Performance following a Total Knee Replacement: A Randomized Controlled Trial. Appl Sci-Basel. 2023 Jan;13(2). PMID: WOS:000914278400001. doi: 10.3390/app13020894.

19.	Zhao R, Cheng L, Zheng Q, Lv Y, Wang YM, Ni M, et al. A Smartphone Application-Based Remote Rehabilitation System for Post-Total Knee Arthroplasty Rehabilitation: A Randomized Controlled Trial. J Arthroplasty. 2024 Mar;39(3):575-81.e8. PMID: 37572720. doi: 10.1016/j.arth.2023.08.019.

20.	Nuevo M, Rodríguez-Rodríguez D, Jauregui R, Fabrellas N, Zabalegui A, Conti M, et al. Telerehabilitation following fast-track total knee arthroplasty is effective and safe: a randomized controlled trial with the ReHub® platform. Disabil Rehabil. 2024 Jun;46(12):2629-39. PMID: 37403684. doi: 10.1080/09638288.2023.2228689.

21.	Pua YH, Yeo SJ, Clark RA, Tan BY, Haines T, Bettger JP, et al. Cost and outcomes of Hospital-based Usual cAre versus Tele-monitor self-directed Rehabilitation (HUATR) in patients with total knee arthroplasty: A randomized, controlled, non-inferiority trial. Osteoarthritis Cartilage. 2024 May;32(5):601-11. PMID: 38049030. doi: 10.1016/j.joca.2023.11.017.

22.	Bradbury TL, McConnell MJ, Whitacre D, Naylor BH, Gibson BT, DeCook CA. A Remote Physical Therapy Program Demonstrates Similar Outcomes Compared to In-Person, Supervised Physical Therapy After Same-Day Discharge Total Knee Arthroplasty: A Randomized Clinical Trial. J Arthroplasty. 2024 Nov;39(11):2725-30.e4. PMID: 38768768. doi: 10.1016/j.arth.2024.05.040.

23.	Pérez-Maletzki J, Roig-Casasús S, Díaz-Díaz B, Martínez-Garrido I, Hernández-Guillén D, Blasco JM. A chatbot tool on a smart mobile device to promote adherence to home rehabilitation after total knee replacement: A randomized controlled trial. Physiotherapy. 2026 Sep;132:101872. PMID: 41934738. doi: 10.1016/j.physio.2025.101872.

24.	Jin C, Feng YJ, Ni YJ, Shan ZL. Clinical Study on Rehabilitation Methods Based on Virtual Reality Technology for Early Functional Exercise After TKA. Chinese Journal of Control of Endemic Diseases. 2017;32(07):779-80.

25.	Sun JW, Sun Q. Effect of home tele-rehabilitation guidance on physical function of patients after TKA. Chinese Journal of Rehabilitation. 2017;32(01):27-9.

26.	Li GW, Li YJ, Zhang DW, Wang Z, Chen T. Application Effects of Virtual Reality Technology in Early Functional Exercise After Total Knee Arthroplasty in Elderly Patients. Chinese Journal of Gerontology. 2018;38(10):2391-3.

27.	Feng YJ, Jin C, Ni YJ, Shan ZL. Effect of virtual reality on functional recovery and psychologicalimprovement after total knee arthroplastyimprovement after total knee arthroplas. Chinese Journal of Bone and Joint Surgery. 2018;11(02):127-32.

28.	Zhao ZX, Wen L, Guo WL, Zhang XD, Jia B. Smart wearable system aided rehabilitation versus conventional functional rehabilitation after total knee arthro⁃plasty. Orthopedic Journal of China. 2018;26(20):1861-6.

29.	Ding F, Zhao ZX, Chen JY. The clinical outcomes of wearable range of motion measuring device in early rehabilitation af-ter total knee arthroplas. Journal of Clinical Orthopaedic. 2019;22(06):693-6.

30.	Zhang JL, Rong GX, Tang Z, Gui BJ. A prospective study on the application of intelligent wearable program in rehabilitation after totalknee arthroplasty. Chinese Journal of Geriatric Orthopaedics and Rehabilitation (Electronic Edition). 2020;6(03):153-8.

31.	Li WC, Song KP, Huang TW, Chen XL, Liu QL, Zhao L, et al. Effect of mobile application rehabilitation guidance on patients after total knee arthroplasty. Chinese Journal of Joint Surgery(Electronic Edition). 2021;15(05):547-53.

32.	Zhou YY, Liu Y, Yuan P, Mao YH. Application value of a wearable precise rehabilitationsystem in treatment of elderly patientsafter total knee arthroplas. Journal of Clinical Medicine in Practice. 2022;26(17):1-4+9.

33.	Li YS. Study on the efficacy and safety oftelerehabilitation strategy based on WeChat intotal knee arthroplasty [Master's Degree]: Sichuan University; 2023.

34.	Li B. Research on rehabilitation prescriptions for early postoperativetotal knee arthroplasty by balance disorder rehabilitation robot [Master's Degree]: Zunyi Medical University; 2023.

35.	Ma YJ. Application of lower limb intelligent weight-bearingrehabilitation robot in early rehabilitation after total kneearthroplasty [Master's Degree]: Yanan University; 2023.

36.	Fan B, Liu L, Xie CQ, Jiang Y, Li P. Efficacy of robot-assisted balance training in functional reconstructionafter total knee arthroplast. Chinese Journal of Robotic Surgery. 2025;6(10):1727-33.
