# Supplementary material for: Effectiveness of Digital Health Interventions on Rehabilitation Outcomes in Patients After Total Knee Arthroplasty: Systematic Review and Network Meta-Analysis
Source: J Med Internet Res. 2026 Nov 13;28:e101277. doi: 10.2196/101277 (PMC13762687; doi:10.2196/101277)
Supplement: Supplementary file 3 [file jmir-v28-e101277-s003.docx]

**Multimedia Appendix 4. Prediction interval.**

| **(A)**   | **(B)**   | **(C)**   |
| --- | --- | --- |
| **(D)**   | **(E)**   | |

**Figure S4.** Forest plot comparing confidence intervals and prediction intervals. A, Pain; B, Active Flexion Range of Motion (ROM); C, Western Ontario and McMaster Universities Osteoarthritis Index (WOMAC); D, Hospital for Special Surgery Knee Score (HSS); E, Timed "Up and Go" (TUG) ; CI=confidence interval.
