# Supplementary material for: Effectiveness of Digital Health Interventions on Rehabilitation Outcomes in Patients After Total Knee Arthroplasty: Systematic Review and Network Meta-Analysis
Source: J Med Internet Res. 2026 Nov 13;28:e101277. doi: 10.2196/101277 (PMC13762687; doi:10.2196/101277)
Supplement: Supplementary file 4 [file jmir-v28-e101277-s004.docx]

**Multimedia Appendix 5. Subgroup analysis.**

**(A)**

**(B)**

**(C)**

**(D)**

**(E)**

**Figure S5.1.** Figure S2. Forest plot of subgroup analysis by intervention type. A, Pain; B, Active Flexion Range of Motion (ROM); C, Western Ontario and McMaster Universities Osteoarthritis Index (WOMAC); D, Hospital for Special Surgery Knee Score (HSS); E, Timed "Up and Go" (TUG); VR, virtual reality; AR, augmented reality; VRT, VR-based telerehabilitation; MAT, mobile application-based telerehabilitation; IVT, internet video conference-based telerehabilitation; IRT, intelligent rehabilitation system-based telerehabilitation; TFT, telephone follow-up-based telerehabilitation; RR, rehabilitation robots.

| **(A)**   | **(B)**   | **(C)**   |
| --- | --- | --- |
| **(D)**   | **(E)**   | |

**Figure S5.2.** Forest plot of subgroup analysis by technology type (wearable sensors, cameras, and web platforms) within the intelligent rehabilitation system-based telerehabilitation (IRT) node. A, Pain; B, Active Flexion ROM; C, WOMAC; D, HSS; E, TUG.

| **(A)** | **(B)**   | **(C)**   |
| --- | --- | --- |
| **(D)**   | **(E)**   | |

**Figure S5.3.** Forest plots of subgroup analysis by treatment dosage matching across outcomes. A, Pain; B, Active Flexion ROM; C, WOMAC; D, HSS; E, TUG.

| **(A)**   | **(B)**   | **(C)**   |
| --- | --- | --- |
| **(D)**   | **(E)**   | |

**Figure S5.4.** Forest plots of subgroup analysis by assessment time window across outcomes. A, Pain; B, Active Flexion ROM; C, WOMAC; D, HSS; E, TUG.

| **(A)**   | **(B)**   | **(C)**   |
| --- | --- | --- |
| **(D)**   | **(E)**   | |

**Figure S5.5.** Forest plots of subgroup analysis by language of publication (Chinese vs non-Chinese) for pain. A, Pain; B, Active Flexion ROM; C, WOMAC; D, HSS; E, TUG.
