# Supplementary material for: Effectiveness of Digital Health Interventions on Rehabilitation Outcomes in Patients After Total Knee Arthroplasty: Systematic Review and Network Meta-Analysis
Source: J Med Internet Res. 2026 Nov 13;28:e101277. doi: 10.2196/101277 (PMC13762687; doi:10.2196/101277)
Supplement: Supplementary file 5 [file jmir-v28-e101277-s005.docx]

**Multimedia Appendix 6. Egger test.**

**Table S6.** Egger test.

| Outcomes | Std_Eff | Coefficient | Std. err | *t* | *P>\|t\|* | (95% conf. interval) |
| --- | --- | --- | --- | --- | --- | --- |
| Pain | Slope | -.0579055 | .2515512 | -0.23 | 0.820 | -.5795908 .4637798 |
|  | Bias | -.6065503 | .8379655 | -0.72 | 0.447 | -2.344384 1.131284 |
| ROM | slope | 6.832989 | 1.017148 | 6.72 | 0.000 | 4.728859 8.937119 |
|  | bias | -1.360284 | .7241295 | -1.88 | 0.073 | -2.85826 .1376923 |
| WOMAC | slope | -1.272759 | 1.670702 | -0.76 | 0.464 | -4.995314 2.449796 |
|  | bias | -1.022437 | 1.5002 | -0.68 | 0.511 | -4.365091 2.320217 |
| HSS | slope | 7.566217 | 3.774295 | 2.00 | 0.080 | -1.137324 16.26976 |
|  | bias | -1.248224 | 3.057906 | -0.41 | 0.694 | -8.299767 5.803319 |
| TUG | slope | -.8260606 | .6795444 | -1.22 | 0.250 | -2.321728 .6696067 |
|  | bias | -1.206423 | 1.238386 | -0.97 | 0.351 | -3.932093 1.519248 |
