# Supplementary material for: Effectiveness of Digital Health Interventions on Rehabilitation Outcomes in Patients After Total Knee Arthroplasty: Systematic Review and Network Meta-Analysis
Source: J Med Internet Res. 2026 Nov 13;28:e101277. doi: 10.2196/101277 (PMC13762687; doi:10.2196/101277)
Supplement: Supplementary file 6 [file jmir-v28-e101277-s006.docx]

**Multimedia Appendix 7. Sensitivity analyses.**

| **(A)**   | **(B)**   | **(C)**   |
| --- | --- | --- |
| **(D)**   | **(E)**   | |

**Figure S7.1.** Leave-one-out sensitivity analysis. A, Pain; B, Active Flexion Range of Motion (ROM); C, Western Ontario and McMaster Universities Osteoarthritis Index (WOMAC); D, Hospital for Special Surgery Knee Score (HSS); E, Timed "Up and Go" (TUG); CI=confidence interval.

| **(A)**   | **(B)**   | **(C)**   |
| --- | --- | --- |
| **(D)**   | **(E)**   | |

**Figure S7.2.** Network evidence plot for the sensitivity analysis using alternative node definitions. A, Pain; B, Active Flexion Active Flexion ROM; C, WOMAC; D, HSS; E, TUG; IDT, Immersive Digital Technology; TR, Telerehabilitation; RR, Rehabilitation Robot; CR, Conventional Rehabilitation.

| **(A)**   | **(B)**   | **(C)**   |
| --- | --- | --- |
| **(D)**   | **(E)**   | |

**Figure S7.3.** SUCRA ranking plot for the sensitivity analysis with alternative node definitions. A, Pain; B, Active Flexion ROM; C, WOMAC; D, HSS; E, TUG; IDT, Immersive Digital Technology; TR, Telerehabilitation; RR, Rehabilitation Robot; CR, Conventional Rehabilitation.

| **(A)**   | **(B)**   | **(C)**   |
| --- | --- | --- |
| **(D)**   | **(E)**   | |

**Figure S7.4.** Rankograms for the sensitivity analysis using the alternative node classification. A, Pain; B, Active Flexion ROM; C, WOMAC; D, HSS; E, TUG; IDT, Immersive Digital Technology; TR, Telerehabilitation; RR, Rehabilitation Robot; CR, Conventional Rehabilitation

| **(A)**   | **(B)**   | **(C)**   |
| --- | --- | --- |
| **(D)**   | **(E)**   | |

**Figure S7.5.** Forest plot for pairwise comparisons in the sensitivity analysis based on the alternative node classification. A, Pain; B, Active Flexion ROM; C, WOMAC; D, HSS; E, TUG; IDT, Immersive Digital Technology; TR, Telerehabilitation; RR, Rehabilitation Robot; CR, Conventional Rehabilitation.

| **(A)**   | **(B)**   | **(C)**   |
| --- | --- | --- |
| **(D)**   | **(E)**   |  |

**Figure S7.6.** Comparison-adjusted funnel plot for the sensitivity analysis using the alternative node classification. A, Pain; B, Active Flexion ROM; C, WOMAC; D, HSS; E, TUG; IDT, Immersive Digital Technology; TR, Telerehabilitation; RR, Rehabilitation Robot; CR, Conventional Rehabilitation.

| **(A)**   | **(B)**   | **(C)**   |
| --- | --- | --- |
| **(D)**   | | |

**Figure S7.7.** Forest plot of treatment effects for the sensitivity analysis excluding studies at high risk of bias. A, Pain; B, Active Flexion ROM; C, WOMAC; D, TUG.

| **(A)**   | **(B)**   | **(C)**   |
| --- | --- | --- |
| **(D)**   | **(E)**   | |

**Figure S7.8.** Forest plots of the sensitivity analysis excluding unpublished theses or non-peer-reviewed sources. A, Pain; B, Active Flexion ROM; C, WOMAC; D, HSS; E, TUG.

| **(A)**   | **(B)**   | **(C)**   |
| --- | --- | --- |
| **(D)**   | | |

**Figure S7.9.** Forest plots of the sensitivity analysis excluding very small trials for pain. A, Pain; B, Active Flexion ROM; C, WOMAC; D, TUG.

| **(A)**   | **(B)**   | **(C)**   |
| --- | --- | --- |
| **(D)**   | | |

**Figure S7.10.** Forest plots of the sensitivity analysis excluding studies that directly reported change-score standard deviations. A, Pain; B, Active Flexion ROM; C, WOMAC; D, TUG.

| **(A)**   | **(B)**   | **(C)**   |
| --- | --- | --- |
| **(D)**   | **(E)**   | |

**Figure S7.11.** Forest plots of the sensitivity analysis using final values instead of change scores. A, Pain; B, Active Flexion ROM; C, WOMAC; D, HSS; E, TUG.
