# Supplementary material for: Effectiveness of Digital Health Interventions on Rehabilitation Outcomes in Patients After Total Knee Arthroplasty: Systematic Review and Network Meta-Analysis
Source: J Med Internet Res. 2026 Nov 13;28:e101277. doi: 10.2196/101277 (PMC13762687; doi:10.2196/101277)
Supplement: Supplementary file 7 [file jmir-v28-e101277-s007.docx]

**Multimedia Appendix 8. Transitivity assumption.**

| **(A)**   | **(B)**   | **(C)**   |
| --- | --- | --- |
| **(D)**   | **(E)**   | |

**Figure S8.1.** Boxplot of participant age across treatment nodes for assessing the transitivity assumption. A, Pain; B, Active Flexion Range of Motion (ROM); C, Western Ontario and McMaster Universities Osteoarthritis Index (WOMAC); D, Hospital for Special Surgery Knee Score (HSS); E, Timed "Up and Go" (TUG); VR, virtual reality; VRT, VR-based telerehabilitation; MAT, mobile application-based telerehabilitation; IVT, internet video conference-based telerehabilitation; IRT, intelligent rehabilitation system-based telerehabilitation; TFT, telephone follow-up-based telerehabilitation; RR, rehabilitation robots.

| **(A)**   | **(B)**   | **(C)**   |
| --- | --- | --- |
| **(D)**   | **(E)**   | |

**Figure S8.2.** Boxplot of sample size across treatment nodes for assessing the transitivity assumption. A, Pain; B, Active Flexion ROM; C, WOMAC; D, HSS; E, TUG; VR, virtual reality; VRT, VR-based telerehabilitation; MAT, mobile application-based telerehabilitation; IVT, internet video conference-based telerehabilitation; IRT, intelligent rehabilitation system-based telerehabilitation; TFT, telephone follow-up-based telerehabilitation; RR, rehabilitation robots.

| **(A)**   | **(B)**   | **(C)**   |
| --- | --- | --- |
| **(D)**   | **(E)**   | |

**Figure S8.3.** Boxplot of intervention duration across treatment nodes for assessing the transitivity assumption. A, Pain; B, Active Flexion ROM; C, WOMAC; D, HSS; E, TUG; VR, virtual reality; VRT, VR-based telerehabilitation; MAT, mobile application-based telerehabilitation; IVT, internet video conference-based telerehabilitation; IRT, intelligent rehabilitation system-based telerehabilitation; TFT, telephone follow-up-based telerehabilitation; RR, rehabilitation robots.

| **(A)**   | **(B)**   | **(C)**   |
| --- | --- | --- |
| **(D)**   | **(E)**   |  |

**Figure S8.4.** Boxplot of baseline characteristics across treatment nodes for assessing the transitivity assumption. A, Pain; B, Active Flexion ROM; C, WOMAC; D, HSS; E, TUG; VR, virtual reality; VRT, VR-based telerehabilitation; MAT, mobile application-based telerehabilitation; IVT, internet video conference-based telerehabilitation; IRT, intelligent rehabilitation system-based telerehabilitation; TFT, telephone follow-up-based telerehabilitation; RR, rehabilitation robots.
