# Supplementary material for: Effectiveness of Digital Health Interventions on Rehabilitation Outcomes in Patients After Total Knee Arthroplasty: Systematic Review and Network Meta-Analysis
Source: J Med Internet Res. 2026 Nov 13;28:e101277. doi: 10.2196/101277 (PMC13762687; doi:10.2196/101277)
Supplement: Supplementary file 8 [file jmir-v28-e101277-s008.docx]

**Multimedia Appendix 9. CINeMA Assessment.**

**Figure S9.1.** Risk of bias contribution by intervention group in pain score. VR, virtual reality; VRT, VR-based telerehabilitation; MAT, mobile application-based telerehabilitation; IVT, internet video conference-based telerehabilitation; IRT, intelligent rehabilitation system-based telerehabilitation; TFT, telephone follow-up-based telerehabilitation; RR, rehabilitation robots; CR, conventional rehabilitation.

**Figure S9.2.** Overall risk of bias by treatment comparison in pain score. VR, virtual reality; VRT, VR-based telerehabilitation; MAT, mobile application-based telerehabilitation; IVT, internet video conference-based telerehabilitation; IRT, intelligent rehabilitation system-based telerehabilitation; TFT, telephone follow-up-based telerehabilitation; RR, rehabilitation robots; CR, conventional rehabilitation.

**Table S9.1.** CINeMA results of pain score.

| Comparison | Number of studies | Within-study bias | Reporting bias | Indirectness | Imprecision | Heterogeneity | Incoherence | Confidence rating |
| --- | --- | --- | --- | --- | --- | --- | --- | --- |
| CR:IRT | 5 | Some concerns | Low risk | No concerns | No concerns | Major concerns | No concerns | Very low |
| CR:IVT | 1 | No concerns | Low risk | No concerns | Major concerns | No concerns | No concerns | Low |
| CR:MAT | 4 | Some concerns | Low risk | No concerns | Major concerns | No concerns | No concerns | Very low |
| CR:RR | 3 | Some concerns | Low risk | No concerns | No concerns | Major concerns | No concerns | Very low |
| CR:TFT | 1 | Some concerns | Low risk | No concerns | Major concerns | No concerns | No concerns | Very low |
| CR:VR | 7 | Some concerns | Low risk | No concerns | No concerns | Major concerns | No concerns | Very low |
| CR:VRT | 3 | Some concerns | Low risk | No concerns | Major concerns | No concerns | No concerns | Very low |
| IRT:IVT | 0 | No concerns | Low risk | No concerns | Major concerns | No concerns | Major concerns | Very low |
| IRT:MAT | 0 | Some concerns | Low risk | No concerns | No concerns | No concerns | Major concerns | Very low |
| IRT:RR | 0 | Some concerns | Low risk | No concerns | No concerns | No concerns | Major concerns | Very low |
| IRT:TFT | 0 | Some concerns | Low risk | No concerns | Major concerns | No concerns | Major concerns | Very low |
| IRT:VR | 0 | Some concerns | Low risk | No concerns | No concerns | No concerns | Major concerns | Very low |
| IRT:VRT | 0 | Some concerns | Low risk | No concerns | No concerns | Major concerns | Major concerns | Very low |
| IVT:MAT | 0 | No concerns | Low risk | No concerns | Major concerns | No concerns | Major concerns | Very low |
| IVT:RR | 0 | Some concerns | Low risk | No concerns | Major concerns | No concerns | Major concerns | Very low |
| IVT:TFT | 0 | Some concerns | Low risk | No concerns | Major concerns | No concerns | Major concerns | Very low |
| IVT:VR | 0 | No concerns | Low risk | No concerns | Major concerns | No concerns | Major concerns | Very low |
| IVT:VRT | 0 | No concerns | Low risk | No concerns | Major concerns | No concerns | Major concerns | Very low |
| MAT:RR | 0 | Some concerns | Low risk | No concerns | Major concerns | No concerns | Major concerns | Very low |
| MAT:TFT | 0 | Some concerns | Low risk | No concerns | Major concerns | No concerns | Major concerns | Very low |
| MAT:VR | 0 | Some concerns | Low risk | No concerns | Major concerns | No concerns | Major concerns | Very low |
| MAT:VRT | 0 | Some concerns | Low risk | No concerns | Major concerns | No concerns | Major concerns | Very low |
| RR:TFT | 0 | Some concerns | Low risk | No concerns | Major concerns | No concerns | Major concerns | Very low |
| RR:VR | 0 | Some concerns | Low risk | No concerns | Major concerns | No concerns | Major concerns | Very low |
| RR:VRT | 0 | Some concerns | Low risk | No concerns | Major concerns | No concerns | Major concerns | Very low |
| TFT:VR | 0 | Some concerns | Low risk | No concerns | Major concerns | No concerns | Major concerns | Very low |
| TFT:VRT | 0 | Some concerns | Low risk | No concerns | Major concerns | No concerns | Major concerns | Very low |
| VR:VRT | 0 | Some concerns | Low risk | No concerns | Major concerns | No concerns | Major concerns | Very low |

VR, virtual reality; VRT, VR-based telerehabilitation; MAT, mobile application-based telerehabilitation; IVT, internet video conference-based telerehabilitation; IRT, intelligent rehabilitation system-based telerehabilitation; TFT, telephone follow-up-based telerehabilitation; RR, rehabilitation robots; CR, conventional rehabilitation.

**Figure S9.3.** Risk of bias contribution by intervention group in ROM. VR, virtual reality; VRT, VR-based telerehabilitation; MAT, mobile application-based telerehabilitation; IVT, internet video conference-based telerehabilitation; IRT, intelligent rehabilitation system-based telerehabilitation; TFT, telephone follow-up-based telerehabilitation; RR, rehabilitation robots; CR, conventional rehabilitation.

**Figure S9.4.** Overall risk of bias by treatment comparison in ROM. VR, virtual reality; VRT, VR-based telerehabilitation; MAT, mobile application-based telerehabilitation; IVT, internet video conference-based telerehabilitation; IRT, intelligent rehabilitation system-based telerehabilitation; TFT, telephone follow-up-based telerehabilitation; RR, rehabilitation robots; CR, conventional rehabilitation.

**Table S9.2.** CINeMA results of ROM.

| Comparison | Number of studies | Within-study bias | Reporting bias | Indirectness | Imprecision | Heterogeneity | Incoherence | Confidence rating |
| --- | --- | --- | --- | --- | --- | --- | --- | --- |
| CR:IRT | 7 | Some concerns | Low risk | No concerns | Major concerns | No concerns | No concerns | Very low |
| CR:IVT | 2 | No concerns | Low risk | No concerns | Major concerns | No concerns | No concerns | Low |
| CR:MAT | 3 | No concerns | Low risk | No concerns | No concerns | No concerns | No concerns | High |
| CR:RR | 5 | Some concerns | Low risk | No concerns | No concerns | No concerns | No concerns | Moderate |
| CR:TFT | 2 | Some concerns | Low risk | No concerns | Major concerns | No concerns | No concerns | Very low |
| CR:VR | 4 | Some concerns | Low risk | No concerns | No concerns | No concerns | No concerns | Moderate |
| CR:VRT | 2 | No concerns | Low risk | No concerns | Major concerns | No concerns | No concerns | Low |
| IRT:IVT | 0 | Some concerns | Low risk | No concerns | Major concerns | No concerns | Major concerns | Very low |
| IRT:MAT | 0 | Some concerns | Low risk | No concerns | No concerns | Major concerns | Major concerns | Very low |
| IRT:RR | 0 | Some concerns | Low risk | No concerns | No concerns | No concerns | Major concerns | Very low |
| IRT:TFT | 0 | Some concerns | Low risk | No concerns | Major concerns | No concerns | Major concerns | Very low |
| IRT:VR | 0 | Some concerns | Low risk | No concerns | No concerns | Major concerns | Major concerns | Very low |
| IRT:VRT | 0 | Some concerns | Low risk | No concerns | Major concerns | No concerns | Major concerns | Very low |
| IVT:MAT | 0 | No concerns | Low risk | No concerns | No concerns | Major concerns | Major concerns | Very low |
| IVT:RR | 0 | No concerns | Low risk | No concerns | No concerns | No concerns | Major concerns | Low |
| IVT:TFT | 0 | Some concerns | Low risk | No concerns | Major concerns | No concerns | Major concerns | Very low |
| IVT:VR | 0 | No concerns | Low risk | No concerns | No concerns | Major concerns | Major concerns | High |
| IVT:VRT | 0 | No concerns | Low risk | No concerns | Major concerns | No concerns | Major concerns | Very low |
| MAT:RR | 0 | No concerns | Low risk | No concerns | Major concerns | No concerns | Major concerns | Very low |
| MAT:TFT | 0 | Some concerns | Low risk | No concerns | Major concerns | No concerns | Major concerns | Very low |
| MAT:VR | 0 | No concerns | Low risk | No concerns | Major concerns | No concerns | Major concerns | Very low |
| MAT:VRT | 0 | No concerns | Low risk | No concerns | Major concerns | No concerns | Major concerns | Very low |
| RR:TFT | 0 | Some concerns | Low risk | No concerns | No concerns | No concerns | Major concerns | Very low |
| RR:VR | 0 | Some concerns | Low risk | No concerns | Major concerns | No concerns | Major concerns | Very low |
| RR:VRT | 0 | Some concerns | Low risk | No concerns | No concerns | Major concerns | Major concerns | Very low |
| TFT:VR | 0 | Some concerns | Low risk | No concerns | Major concerns | No concerns | Major concerns | Very low |
| TFT:VRT | 0 | Some concerns | Low risk | No concerns | Major concerns | No concerns | Major concerns | Very low |
| VR:VRT | 0 | Some concerns | Low risk | No concerns | Major concerns | No concerns | Major concerns | Very low |

VR, virtual reality; VRT, VR-based telerehabilitation; MAT, mobile application-based telerehabilitation; IVT, internet video conference-based telerehabilitation; IRT, intelligent rehabilitation system-based telerehabilitation; TFT, telephone follow-up-based telerehabilitation; RR, rehabilitation robots; CR, conventional rehabilitation.

**Figure S9.5.** Risk of bias contribution by intervention group in WOMAC. VR, virtual reality; MAT, mobile application-based telerehabilitation; IVT, internet video conference-based telerehabilitation; RR, rehabilitation robots; CR, conventional rehabilitation; IRT, intelligent rehabilitation system-based telerehabilitation.

**Figure S9.6.** Overall risk of bias by treatment comparison in WOMAC. VR, virtual reality; MAT, mobile application-based telerehabilitation; IVT, internet video conference-based telerehabilitation; RR, rehabilitation robots; CR, conventional rehabilitation; IRT, intelligent rehabilitation system-based telerehabilitation.

**Table S9.3.** CINeMA results of WOMAC.

| Comparison | Number of studies | Within-study bias | Reporting bias | Indirectness | Imprecision | Heterogeneity | Incoherence | Confidence rating |
| --- | --- | --- | --- | --- | --- | --- | --- | --- |
| CR:IRT | 5 | Some concerns | Low risk | No concerns | Major concerns | No concerns | No concerns | Very low |
| CR:IVT | 1 | No concerns | Low risk | No concerns | No concerns | Major concerns | No concerns | Low |
| CR:MAT | 3 | No concerns | Low risk | No concerns | Major concerns | No concerns | No concerns | Low |
| CR:RR | 1 | Some concerns | Low risk | No concerns | Major concerns | No concerns | No concerns | Very low |
| CR:VR | 3 | Some concerns | Low risk | No concerns | Major concerns | No concerns | No concerns | Very low |
| IRT:IVT | 0 | No concerns | Low risk | No concerns | Major concerns | No concerns | Major concerns | Very low |
| IRT:MAT | 0 | Some concerns | Low risk | No concerns | Major concerns | No concerns | Major concerns | Very low |
| IRT:RR | 0 | Some concerns | Low risk | No concerns | Major concerns | No concerns | Major concerns | Very low |
| IRT:VR | 0 | Some concerns | Low risk | No concerns | Major concerns | No concerns | Major concerns | Very low |
| IVT:MAT | 0 | No concerns | Low risk | No concerns | Major concerns | No concerns | Major concerns | Very low |
| IVT:RR | 0 | Some concerns | Low risk | No concerns | Major concerns | No concerns | Major concerns | Very low |
| IVT:VR | 0 | Some concerns | Low risk | No concerns | Major concerns | No concerns | Major concerns | Very low |
| MAT:RR | 0 | Some concerns | Low risk | No concerns | Major concerns | No concerns | Major concerns | Very low |
| MAT:VR | 0 | Some concerns | Low risk | No concerns | Major concerns | No concerns | Major concerns | Very low |
| RR:VR | 0 | Some concerns | Low risk | No concerns | Major concerns | No concerns | Major concerns | Very low |

VR, virtual reality; VRT, VR-based telerehabilitation; MAT, mobile application-based telerehabilitation; IVT, internet video conference-based telerehabilitation; IRT, intelligent rehabilitation system-based telerehabilitation; TFT, telephone follow-up-based telerehabilitation; RR, rehabilitation robots; CR, conventional rehabilitation.

**Figure S9.7.** Risk of bias contribution by intervention group in HSS. VR, virtual reality; RR, rehabilitation robots; CR, conventional rehabilitation; IRT, intelligent rehabilitation system-based telerehabilitation.

**Figure S9.8.** Overall risk of bias by treatment comparison in HSS. VR, virtual reality; RR, rehabilitation robots; CR, conventional rehabilitation; IRT, intelligent rehabilitation system-based telerehabilitation.

**Table S9.4.** CINeMA results of HSS.

| Comparison | Number of studies | Within-study bias | Reporting bias | Indirectness | Imprecision | Heterogeneity | Incoherence | Confidence rating |
| --- | --- | --- | --- | --- | --- | --- | --- | --- |
| CR:IRT | 3 | Some concerns | Low risk | No concerns | No concerns | Major concerns | No concerns | Very low |
| CR:RR | 4 | Some concerns | Low risk | No concerns | No concerns | Major concerns | No concerns | Low |
| CR:VR | 3 | Some concerns | Low risk | No concerns | No concerns | No concerns | No concerns | Moderate |
| IRT:RR | 0 | Some concerns | Low risk | No concerns | Major concerns | No concerns | Major concerns | Very low |
| IRT:VR | 0 | Some concerns | Low risk | No concerns | Major concerns | No concerns | Major concerns | Very low |
| RR:VR | 0 | Some concerns | Low risk | No concerns | Major concerns | No concerns | Major concerns | Very low |

VR, virtual reality; RR, rehabilitation robots; CR, conventional rehabilitation; IRT, intelligent rehabilitation system-based telerehabilitation.

**Figure S9.9.** Risk of bias contribution by intervention group in TUG. VR, virtual reality; VRT, VR-based telerehabilitation; MAT, mobile application-based telerehabilitation; IVT, internet video conference-based telerehabilitation; IRT, intelligent rehabilitation system-based telerehabilitation; RR, rehabilitation robots; CR, conventional rehabilitation.

**Figure S9.8.** Overall risk of bias by treatment comparison in TUG. VR, virtual reality; VRT, VR-based telerehabilitation; MAT, mobile application-based telerehabilitation; IVT, internet video conference-based telerehabilitation; IRT, intelligent rehabilitation system-based telerehabilitation; RR, rehabilitation robots; CR, conventional rehabilitation.

**Table S9.5.** CINeMA results of TUG.

| Comparison | Number of studies | Within-study bias | Reporting bias | Indirectness | Imprecision | Heterogeneity | Incoherence | Confidence rating |
| --- | --- | --- | --- | --- | --- | --- | --- | --- |
| CR:IRT | 3 | Some concerns | Low risk | No concerns | Major concerns | No concerns | No concerns | Very low |
| CR:IVT | 2 | Some concerns | Low risk | No concerns | Major concerns | No concerns | No concerns | Very low |
| CR:MAT | 2 | Some concerns | Low risk | No concerns | No concerns | Major concerns | No concerns | Very low |
| CR:RR | 2 | Some concerns | Low risk | No concerns | Major concerns | No concerns | No concerns | Very low |
| CR:VR | 2 | Some concerns | Low risk | No concerns | Major concerns | No concerns | No concerns | Very low |
| CR:VRT | 2 | Some concerns | Low risk | No concerns | No concerns | Major concerns | No concerns | Very low |
| IRT:IVT | 0 | Some concerns | Low risk | No concerns | Major concerns | No concerns | Major concerns | Very low |
| IRT:MAT | 0 | Some concerns | Low risk | No concerns | Major concerns | No concerns | Major concerns | Very low |
| IRT:RR | 0 | Some concerns | Low risk | No concerns | Major concerns | No concerns | Major concerns | Very low |
| IRT:VR | 0 | Some concerns | Low risk | No concerns | Major concerns | No concerns | Major concerns | Very low |
| IRT:VRT | 0 | Some concerns | Low risk | No concerns | No concerns | Major concerns | Major concerns | Very low |
| IVT:MAT | 0 | Some concerns | Low risk | No concerns | Major concerns | No concerns | Major concerns | Very low |
| IVT:RR | 0 | Some concerns | Low risk | No concerns | Major concerns | No concerns | Major concerns | Very low |
| IVT:VR | 0 | Some concerns | Low risk | No concerns | Major concerns | No concerns | Major concerns | Very low |
| IVT:VRT | 0 | Some concerns | Low risk | No concerns | Major concerns | No concerns | Major concerns | Very low |
| MAT:RR | 0 | Some concerns | Low risk | No concerns | Major concerns | No concerns | Major concerns | Very low |
| MAT:VR | 0 | Some concerns | Low risk | No concerns | Major concerns | No concerns | Major concerns | Very low |
| MAT:VRT | 0 | Some concerns | Low risk | No concerns | Major concerns | No concerns | Major concerns | Very low |
| RR:VR | 0 | Some concerns | Low risk | No concerns | Major concerns | No concerns | Major concerns | Very low |
| RR:VRT | 0 | Some concerns | Low risk | No concerns | Major concerns | No concerns | Major concerns | Very low |
| VR:VRT | 0 | Some concerns | Low risk | No concerns | Major concerns | No concerns | Major concerns | Very low |

VR, virtual reality; VRT, VR-based telerehabilitation; MAT, mobile application-based telerehabilitation; IVT, internet video conference-based telerehabilitation; IRT, intelligent rehabilitation system-based telerehabilitation; RR, rehabilitation robots; CR, conventional rehabilitation.
